# Reduced Retinoic Acid Signaling During Gastrulation Induces Developmental Microcephaly

**DOI:** 10.3389/fcell.2022.844619

**Published:** 2022-03-14

**Authors:** Michal Gur, Liat Bendelac-Kapon, Yehuda Shabtai, Graciela Pillemer, Abraham Fainsod

**Affiliations:** Department of Developmental Biology and Cancer Research, Institute for Medical Research Israel-Canada, Faculty of Medicine, The Hebrew University of Jerusalem, Jerusalem, Israel

**Keywords:** retinoic acid biosynthesis, embryo development, Xenopus embryo, CRISPR/Cas9, gene knockdown, Fetal Alcohol Syndrome, prechordal mesoderm, retinaldehyde dehydrogenase

## Abstract

Retinoic acid (RA) is a central signaling molecule regulating multiple developmental decisions during embryogenesis. Excess RA induces head malformations, primarily by expansion of posterior brain structures at the expense of anterior head regions, i.e., hindbrain expansion. Despite this extensively studied RA teratogenic effect, a number of syndromes exhibiting microcephaly, such as DiGeorge, Vitamin A Deficiency, Fetal Alcohol Syndrome, and others, have been attributed to reduced RA signaling. This causative link suggests a requirement for RA signaling during normal head development in all these syndromes. To characterize this novel RA function, we studied the involvement of RA in the early events leading to head formation in *Xenopus* embryos. This effect was mapped to the earliest RA biosynthesis in the embryo within the gastrula Spemann-Mangold organizer. Head malformations were observed when reduced RA signaling was induced in the endogenous Spemann-Mangold organizer and in the ectopic organizer of twinned embryos. Two embryonic retinaldehyde dehydrogenases, ALDH1A2 (RALDH2) and ALDH1A3 (RALDH3) are initially expressed in the organizer and subsequently mark the trunk and the migrating leading edge mesendoderm, respectively. Gene-specific knockdowns and CRISPR/Cas9 targeting show that RALDH3 is a key enzyme involved in RA production required for head formation. These observations indicate that in addition to the teratogenic effect of excess RA on head development, RA signaling also has a positive and required regulatory role in the early formation of the head during gastrula stages. These results identify a novel RA activity that concurs with its proposed reduction in syndromes exhibiting microcephaly.

## Introduction

Microcephaly is a condition in which the brain fails to achieve its normal size (Abuelo, 2007; Toi et al., 2009; Mochida, 2009; Dyment et al., 2013; Faheem et al., 2015; Duerinckx and Abramowicz, 2018). Besides the wide variation in head size in the human population (Natale and Rajagopalan, 2014), individuals with head circumferences (occipitofrontal) smaller by 3 standard deviations from the population mean (age, sex, and ethnicity matched), exhibit what is known as clinical microcephaly (Martini et al., 2018). These individuals encompass up to 0.1% of the human population and most of them suffer from significant intellectual disabilities (Abuelo, 2007). Microcephaly can be subdivided into primary, if developed during embryogenesis and present at birth, or secondary, if developed after birth, but this classification is not universally accepted. To date, Online Mendelian Inheritance in Man (OMIM.org) lists close to a thousand genes, diseases, or syndromes associated with microcephaly in addition to numerous environmental factors that can induce this condition. To elucidate the etiology of primary or developmental microcephaly we need to achieve a better understanding of the signals and processes that regulate the induction, patterning, and differentiation of the rostral neuroectoderm and subsequently the forebrain in the embryo (Abuelo, 2007; Mochida, 2009; Toi et al., 2009; Dyment et al., 2013; Faheem et al., 2015; Duerinckx and Abramowicz, 2018) which in turn will affect the size of the head (Koyabu et al., 2014; Ranke et al., 2015; Martini et al., 2018). In *Xenopus* embryos, the group of cells responsible for anterior neuroectoderm induction and patterning, the head organizer, forms as part of the Spemann-Mangold organizer (Spemann and Mangold, 1924; Inui et al., 2012; Kiecker and Lumsden, 2012; Yanagi et al., 2015). Very early during embryogenesis, the leading edge mesendoderm (LEM)/prechordal mesoderm (PCM) cells migrate to the rostral region beneath the prospective cranial neuroectoderm, and at this position, they will perform their inductive and patterning functions (Kaneda and Motoki, 2012; Huang and Winklbauer, 2018).

In humans, *in utero* exposure to alcohol (ethanol, EtOH) is the environmental disturbance that induces Fetal Alcohol Spectrum Disorder (FASD). Individuals suffering from the severe form of FASD, Fetal Alcohol Syndrome (FAS), suffer from a multitude of developmental malformations including microcephaly (Roussotte et al., 2012; Gautam et al., 2015; Popova et al., 2016; Del Campo and Jones, 2017; Jarmasz et al., 2017; Treit et al., 2017; Petrelli et al., 2019). In FAS, cognitive disabilities, behavioral and social problems, reduced executive functioning, and social withdrawal accompany the microcephaly (Niccols, 2007; Spohr and Steinhausen, 2008; Guerri et al., 2009; Gautam et al., 2014; Popova et al., 2016). In recent years, we established and characterized a *Xenopus*-based experimental model that recapitulates many of the developmental malformations characteristic of FAS following alcohol exposure, including microcephaly (Yelin et al., 2005; Yelin et al., 2007; Kot-Leibovich and Fainsod, 2009; Fainsod and Kot-Leibovich, 2018; Shabtai et al., 2018). We have shown that many of the developmental malformations arising from embryonic alcohol exposure (EAE) are the result of reduced retinoic acid (RA) signaling (Yelin et al., 2005; Kot-Leibovich and Fainsod, 2009; Shabtai et al., 2018; Shabtai and Fainsod, 2018; Fainsod et al., 2020). During early embryogenesis, alcohol clearance and Vitamin A (retinol, ROL) metabolism are performed in part by the same enzymes or enzyme families (Crabb et al., 2004; Shabtai and Fainsod, 2018). Biosynthesis of RA from Vitamin A (retinol, ROL) proceeds through two consecutive oxidation steps, the first performed mainly by short-chain dehydrogenase/reductases (SDR) oxidizing ROL to retinaldehyde (RAL), and the subsequent oxidation step from RAL to RA performed by aldehyde dehydrogenases (ALDH) also known as retinaldehyde dehydrogenases (Kedishvili, 2013; Cunningham and Duester, 2015; Shabtai and Fainsod, 2018). EtOH detoxification in the embryo makes use of some of the same enzymes, sometimes redirecting them from their involvement in RA biosynthesis and other metabolic processes (Duester, 1991; Pullarkat, 1991; Kot-Leibovich and Fainsod, 2009; Shabtai et al., 2018; Shabtai and Fainsod, 2018; Fainsod et al., 2020). As it has extensively been shown, RA is crucial for normal embryonic development and tissue homeostasis (Ross et al., 2000; See et al., 2008; Clagett-Dame and Knutson, 2011; Kin Ting Kam et al., 2012; Rhinn and Dollé, 2012; Cunningham and Duester, 2015; Draut et al., 2019; Nolte et al., 2019), and abnormally high or low levels are extremely teratogenic giving rise to a complex and severe set of developmental malformations (Collins and Mao, 1999; Mark et al., 2006; Ghyselinck and Duester, 2019).

It is widely accepted that increased RA levels adversely affect the formation of the head and in particular, inhibit forebrain development by promoting hindbrain expansion resulting in a microcephalic phenotype (Durston et al., 1989; Sive et al., 1989; Koide et al., 2001; Halilagic et al., 2003; Crandall et al., 2011). On the other hand, reduced RA signaling levels have been linked to an increasing number of developmental syndromes that exhibit microcephaly including FAS, DiGeorge, Smith-Magenis, Matthew-Wood, and Vitamin A Deficiency (VAD) Syndromes, and others (Twal et al., 1995; Collins and Mao, 1999; Maden, 2000; Clagett-Dame and DeLuca, 2002; Vermot et al., 2003; Yelin et al., 2005; Kot-Leibovich and Fainsod, 2009; Chassaing et al., 2009; Elsea and Williams, 2011; Shabtai et al., 2018; Fainsod et al., 2020; Fainsod et al., 2022). This link between microcephaly and reduced RA levels suggests that RA signaling is required during early head induction and forebrain establishment. In agreement, *retinaldehyde dehydrogenase 2* (*Raldh2*; *Aldh1a2*) mutants die very early during development exhibiting malformations of the anterior head region (Niederreither et al., 1999; Begemann et al., 2001; Perz-Edwards et al., 2001; Halilagic et al., 2007) and dorsal knock-down of the RA nuclear receptor, RARα2 results in head truncation and malformations (Shiotsugu et al., 2004).

To further characterize the role of RA signaling during gastrulation and in particular, in the process of head formation, we experimentally manipulated its levels. We show that localized reduction of RA levels within the embryonic organizer results in a high incidence of embryos with abnormally small heads. RA biosynthesis inhibition or signaling knockdown in induced secondary axes also reduces the efficiency of head formation in the twinned embryos. These observations support the suggestion that RA signaling is required for normal head development. In *Xenopus* embryos, the *aldh1a2* gene is initially expressed within the organizer domain but soon thereafter its expression becomes lateralized and is absent from the dorsal midline (Chen et al., 2001). This pattern of expression prompted us to search for additional RA biosynthetic enzymes that could produce the RA required for head induction. *Aldh1a3* (*raldh3*) transcripts were detected in the early organizer (Lupo et al., 2005), and subsequently in the rostrally migrating LEM/PCM cells. We show that *aldh1a3* knockdown with antisense morpholino oligonucleotides or by gene targeting with CRISPR/Cas9 efficiently hampers the formation of head structures, resulting in microcephaly in both the endogenous and induced secondary axes. These results indicate that RA signaling is required in the LEM/PCM cells for normal head formation. Further, we show that ALDH1A3 is the main enzyme involved in producing the RA needed for this process.

## Materials and Methods

### Embryo Culture and Treatments

*Xenopus laevis* embryos were obtained by *in vitro* fertilization, incubated in 0.1% Modified Barth’s Solution and Hepes (MBSH) and staged according to Nieuwkoop and Faber (1967). All experiments were performed after obtaining ethics approval and under the supervision of the Institutional Animal Care and Use Committee (IACUC) of the Hebrew University (Ethics approval no. MD-17-15281-3). Treatments with 4-Diethylaminobenzaldehyde (DEAB, Sigma) or 3,7-Dimethyl-2,6-octadienal (citral, Aldrich), were performed in 0.1% MBSH from the mid-blastula transition (MBT, stage 8) until the desired stage for analysis.

Embryos were injected at the 1-4 cell stage with *in vitro* transcribed capped RNA, expression plasmids, antisense morpholino oligonucleotides, or CRISPR/Cas9 single guide RNAs (sgRNAs). Capped RNAs were prepared using the appropriate RNA polymerase. Cap analog [m7G(5′)ppp(5′)G; New England Biolabs, United States] was added to the reaction mixture using a cap:GTP ratio of 5:1. Expression plasmids were linearized and transcribed as previously described: *wnt8a* (Christian and Moon, 1993), *tALK3* (Graff et al., 1994), *cyp26a1* (Hollemann et al., 1998), *bmp4* (Fainsod et al., 1994), *dkk1* (Glinka et al., 1998). Antisense morpholino oligonucleotides (Table 1) were obtained from Gene Tools LLC (United States).

**TABLE 1:** Primers for PCR expression analysis, genomic amplification and expression knockdown.

Gene	Forward primer	Reverse primer
*RT-qPCR*		
*admp.*S	GCC​TTC​CGA​GCA​AGC​TTA​CTT	CCT​TGT​GGC​AAC​TGT​ATC​TTA​TTT​TTA
*cer1.*S	CTG​GTG​CCA​AGA​TGT​TCT​GGA​A	CGG​CAA​GCA​ATG​GGA​ACA​AGT​A
*chrd.1.*L/S	ACT​GCC​AGG​ACT​GGA​TGG​T	GGC​AGG​ATT​TAG​AGT​TGC​TTC
*cyp26a1.*S	CGA​TTC​CTC​AAG​GTT​TGG​CTT​CA	ATT​TAG​CGG​GTA​GGT​TGT​CCA​CA
*dhrs3.*L	CAG​GCG​CAA​GAA​ATC​CTA​AG	CAA​AGG​CCA​CGT​TAC​AGG​AT
*dkk1.*S	TGC​CTA​CCC​GCT​CTA​CAG​TT	AAC​CAG​AGA​GTT​GCC​GTT​TC
*frzb1.*L	CCA​ATG​CTT​ACT​GTG​CTT​CGT	AGT​GCT​GTG​GTG​GAG​ATG​GT
*gapdh.*S	GCT​CCT​CTC​GCA​AAG​GTC​AT	GGG​CCA​TCC​ACT​GTC​TTC​TG
*gsc.*L/S	TTC​ACC​GAT​GAA​CAA​CTG​GA	TTC​CAC​TTT​TGG​GCA​TTT​TC
*hoxa1.*L	CCG​CTC​ACT​ATA​TCC​ACC​ATT​C	TGG​CAG​GAG​AAC​GAC​AAA​C
*hoxb1.*L	TTG​CCC​CAG​TGC​CAA​TGA​C	TCC​CCC​TCC​AAC​AAC​AAA​CC
*hoxb4.*S	CCA​AGG​ATC​TGT​GCG​TCA​A	GCAGGATGGAGGCGAACT
*myod1.*S	CCC​TGT​TTC​AAT​ACC​TCA​GAC​AT	CGT​GCT​CAT​CCT​CGT​TAT​GG
*otx2.*L	AAG​CCG​CAA​TAT​AGA​AAG​GAA​CA	GGG​ATT​CCT​TGT​CGC​AAT​TAA​TA
*aldh1a1.*L	GAACTTTCCGTTGTTGAT	GAT​AGC​AGT​CAG​TGG​AGT​TTG
*aldh1a2.*L	ATGTTTGCCTGGAAGA	GAGAGCAGTGAGCGGA
*aldh1a3.*L	TAA​AGC​CCT​GTC​TGT​TTC​T	CAT​ACT​CTC​CAA​GTT​CCC​TT
*rdh10.*L/S	CCC​AGA​GTA​ACG​AGG​AGA​CG	ATT​GCA​GCA​CGG​CAG​AAC​T
*szl.*L/S	AAC​AAG​GTC​TGC​TCC​TTC​CA	CTGTGGGTCTGGTCCG
*ventx1.2.*S	AGG​CAG​GAG​TTC​ACA​GGA​AA	TGC​CTG​TTC​CAG​TTT​GCT​T
*Genomic nested PCR*		
Outer genomic PCR		
*aldh1a2.L*	CTG​GGA​TCT​GCT​CAT​TCA​GTG​T	ACG​TTG​ATT​GAT​CCG​TGG​TG
*aldh1a2.S*	CAA​CAA​GTT​CAT​TTT​TCC​TGC​TGA​A	ATA​GGC​AGG​TCT​CTT​GGG​GA
*aldh1a3.L*	TCA​AAG​GAG​AAA​AGG​CTC​AGG​T	TAG​AAA​TTC​ACC​AGC​AGG​AAA​GC
*aldh1a3.S*	ACC​CCA​TAA​AAT​GTG​TGC​TAC​TCT	TTC​TAA​CAG​ACT​GGG​TTG​GGA​TG
Inner genomic PCR		
*aldh1a2.L*	GTG​TAC​CCT​TTG​TAT​GTT​GGC​AT	GCC​ATT​GTG​CTA​CGG​TTT​TG
*aldh1a2.S*	GTC​ACA​CAC​CTT​TCA​GTT​TTT​GG	AGG​TCT​CTT​GGG​GAA​ACT​GTG
*aldh1a3.L*	TTA​CCA​GTG​CAG​GGC​AAC​AG	TCT​AAC​AGA​CTG​GGT​TGG​GGA​T
*aldh1a3.S*	AGA​ACA​ATT​GTG​GGG​CAG​CA	TGC​TGC​ATC​TAG​CTA​TAG​AAA​CCC
*Antisense morpholino oligonucleotides*		
ALDH1A2.L/S	CTA​TTT​TAC​TGG​AAG​TCA​TGT​CTG​G	—
ALDH1A3.L/S	TAG​TGG​TTG​TCA​TGT​TGA​TAG​AGG​C	—
Control MO	CCT​CTT​ACC​TCA​GTT​ACA​ATT​TAT​A	—
*CRISPR (crRNA)*		
*aldh1a2.*L/S	GAA​TGG​ATG​CCT​CAG​AAA​GG	—
*aldh1a3.*L/S	CAG​CAG​TCT​CCC​TCG​GCC​AT	—

### Generation of CRISPant Embryos

For gene-specific single guide RNA design (sgRNA), genomic DNA sequences were selected from Xenbase.org (Nenni et al., 2019) for the L and S homoeologs when present and used CRISPRdirect (Naito et al., 2015) and CRISPRscan (Moreno-Mateos et al., 2015) for target site search. Computational estimation of the sgRNA efficiency was determined using the inDelphi software (Shen et al., 2018; Naert et al., 2020). sgRNA target sequences used are listed in Table 1. For the generation of F0 CRISPant embryos, we injected one-cell stage embryos with Cas9 ribonucleoprotein (RNP) complexes employing the two-RNA component (crRNA:tracrRNA) approach (Hoshijima et al., 2019). Briefly, chemically synthesized and modified for stability (Alt-R) RNAs (crRNA and tracrRNA; IDT, United States) (Table 1) were annealed to generate the double guide complexes (crRNA:tracrRNA), and were incubated (10 min at 37°C) with *S. pyogenes* Cas9 protein (IDT, United States) to generate RNP complexes. Eight nanoliters of the RNP complex solution were injected into the cytoplasm of one-cell stage embryos.

To determine the efficiency of indel induction, genomic DNA was extracted from 5 individual embryos at mid-gastrula (st. 11) or later, employing the GenElute Mammalian Genomic DNA Miniprep Kit (SIGMA). The genomic region containing the CRISPR/Cas9 targeted region was PCR amplified using a nested PCR approach (Table 1) and the size-selected and cleaned product was sequenced. Genome editing efficiency was analyzed by decomposition analysis (Brinkman et al., 2014) using the Synthego ICE algorithm (Hsiau et al., 2018).

### Whole-Mount *in Situ* Hybridization

Whole-mount *in situ* hybridization and double *in situ* hybridization were performed as previously described (Epstein et al., 1997). Probes were prepared by *in vitro* transcription using Digoxigenin or Fluorescein labeling mix (Roche). Double staining was performed by either 5-Bromo-6-chloro-3-indolyl phosphate p-Toluidine salt (Magenta phosphate, Sigma) or BM purple (Roche) for the first probe and 5-Bromo-4-chloro-3-indolyl phosphate p-Toluidine salt (BCIP, Roche) for the second probe. Probes were transcribed as previously described: *pax6* (Li et al., 1997), *ncam1* (Krieg et al., 1989), *muc2* (*XCG-1*) (Sive et al., 1989), *chrd.1* (*chordin*) (Sasai et al., 1994), *gsc* (*goosecoid*) (Cho et al., 1991), *cyp26a1* (Hollemann et al., 1998), *aldh1a2* (*raldh2*) (Chen et al., 2001), *aldh1a3* (*raldh3*) (Lupo et al., 2005), *otx2* (Smith et al., 1993).

### Quantitative Reverse Transcription Real-Time PCR (qPCR)

Total RNA from embryos was extracted with the Aurum™ Total RNA Mini Kit (Bio-Rad) and cDNA was synthesized using the iScript cDNA Synthesis Kit (Bio-Rad). The real-time PCR reactions were performed using the CFX384 Real-Time System (Bio-Rad) and iTaq Universal SYBR Green Supermix (Bio-Rad). Each experiment was repeated at least three independent times and each time the samples were run in triplicate. GAPDH was used as the housekeeping reference gene. The primers used are listed in Table 1.

### ß-Galactosidase Activity Assays

Chemiluminescent quantification of the reporter pRAREhsplacZ plasmid (Rossant et al., 1991) activity was performed using ß-gal Juice Plus (PJK, Germany) as previously described (Yelin et al., 2005). Chemiluminescence activity was measured on a TD-20/20 Luminometer (Turner Designs). *LacZ* RNA was prepared from a clone containing a nuclear localization signal (pSP6nuc ß-gal) in pGEM-3Z (Promega). The staining of embryos for ß-galactosidase activity was performed with 5-bromo-4-chloro-3-indolyl-*β*-D-galactopyranoside (Xgal).

### Statistical Analysis

All statistical comparisons were carried out using the Prism software package (Graph Pad Software Inc., San Diego, CA). Results are given as the mean ± standard error of the mean (SEM). Tests used were the 2-tailed t-test for two-sample comparisons, Dunnett’s (ANOVA) multiple comparisons test, or Fisher test. Differences between means were considered significant at a significance level of *p* < 0.05.

## Results

### Retinoic Acid Signaling Reduction Induces Microcephaly

**FIGURE 1:**
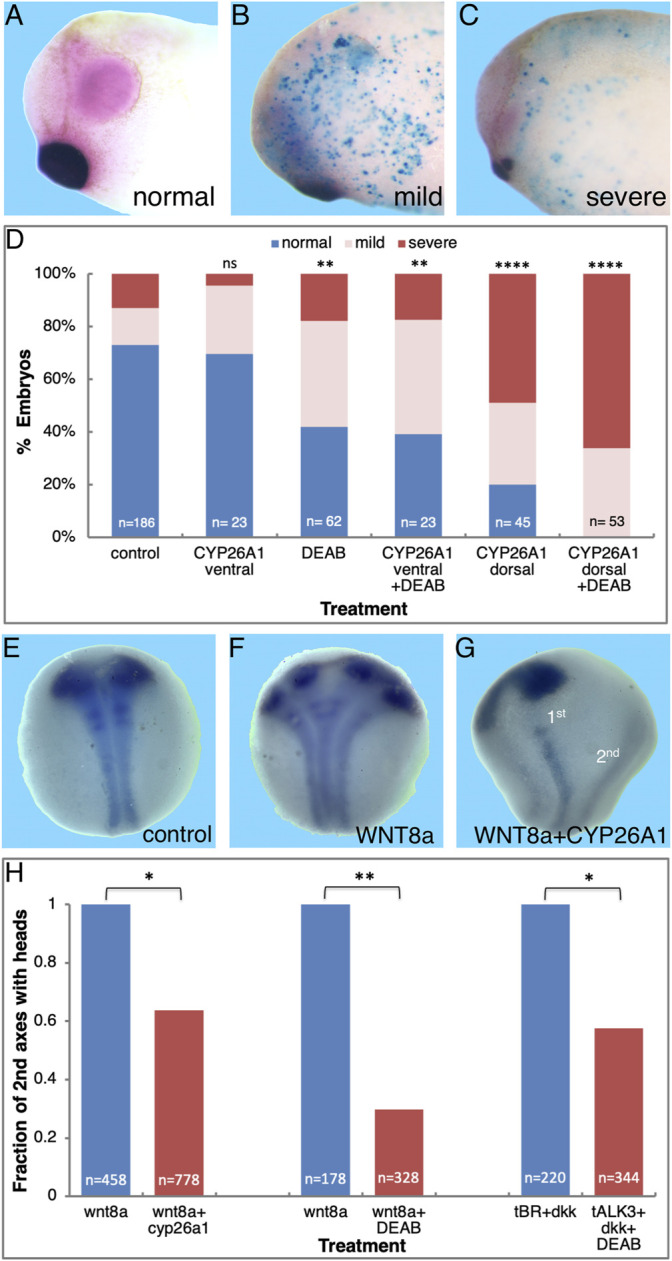
Retinoic acid is required for normal head development. Embryos were injected with RNA encoding CYP26A1 and treated with DEAB to reduce the endogenous levels of RA. **(A)** Control embryo (st. 30) processed for *in situ* hybridization with *pax6* (eyes, pink) and *muc2* (cement gland, purple) specific probes. **(B)** Embryo injected dorsally with *cyp26a1* RNA exhibiting mild microcephaly. Turquoise staining is the *LacZ* lineage tracer. **(C)** Severe microcephaly in an embryo injected with *cyp26a1* mRNA in the dorsal region. **(D)** Frequency of microcephaly induction by combined RA knockdown in embryos injected dorsally or ventrally with *cyp26a1* RNA and treated with DEAB. **(E)** Control st. 14/15 embryo processed for *in situ* hybridization with *pax6* (eyes) and *ncam* (neural plate). **(F)** Embryo ventrally injected with *wnt8a* RNA to induce a secondary axis analyzed for eye and neural plate formation. **(G)** Loss of anterior head structures (eyes, *pax6*) in the induced secondary axis by co-injection of *cyp26a1* RNA with the *wnt8* mRNA. The primary and secondary axes are labeled (1^st^, 2^nd^). **(H)** Inhibition of anterior head formation in twinned embryos induced by ventral *wnt8a* injection or parallel inhibition of BMP (tALK3) and Wnt (DKK1) signaling and reduction of RA levels (DEAB treatment or CYP26A1 overexpression). The overall number of embryos injected or manipulated is shown (*n* =). *, *p* < 0.05; **, *p* < 0.01; ****, 0.0001; ns, not significant.

To support the requirement for RA signaling in the formation of the head, we reduced the endogenous RA levels by localized dorsal or ventral injection of mRNA encoding CYP26A1. The CYP26A1 enzyme is a RA hydroxylase rendering it biologically inactive, thus reducing the activity of this signaling pathway (Catharine Ross and Zolfaghari, 2011). RNA encoding ß-galactosidase (*LacZ*) was co-injected as a lineage tracer to monitor and verify the injection site. To study the effect on anterior head structure formation and to determine whether the embryos exhibit normal, mild, or severe microcephaly, we analyzed the development of the eyes (*pax6*) and cement gland (*muc2*) by *in situ* hybridization (Figures 1A–C). Dorsal CYP26A1 overexpression induced microcephaly with high efficiency (80%), with the majority of the embryos exhibiting severe microcephaly (61.1%; Figures 1B–D). Ventral *cyp26a1* RNA injections resulted in a distribution of head phenotypes similar to control embryos (13.9% very mild microcephaly; Figure 1D). To support the requirement for normal RA signaling levels in head development, we also performed systemic RA biosynthesis inhibition with 4-diethylaminobenzaldehyde (DEAB; 150 μM) (Russo et al., 1988; Morgan et al., 2015; Shabtai et al., 2016). The DEAB treatment induced mild microcephaly in 40.3% of the embryos (Figure 1D). Combined ventral *cyp26A1* mRNA injection with DEAB treatment had no significant additive effect compared to the DEAB treatment alone, increasing only slightly the proportion of mild microcephalic embryos to 43.5%. In contrast, the addition of DEAB to dorsally *cyp26a1* RNA injected embryos increased the incidence of microcephaly to 100% of the embryos (Figure 1D), showing that most of the RA signaling activity required for normal head development is localized dorsally.

Based on the organizer-restricted expression of *aldh1a2* and the activation of RA signaling in the gastrula organizer (Chen et al., 2001; Yelin et al., 2005), we expect activation of RA signaling in supernumerary organizers induced on the ventral side of the embryo. For this reason, we induced secondary axes by either ventral activation of Wnt/ß-catenin signaling (*wnt8a* RNA injection) (Sokol et al., 1991), or inhibition of BMP signaling by *smad6* (Marom et al., 2005) or a dominant negative BMP receptor (tALK3) (Graff et al., 1994) mRNA injection. Activation of RA signaling in the secondary organizer was monitored using the RA reporter pRAREhspLacZ (RAREZ) plasmid (Rossant et al., 1991; Yelin et al., 2005) which was co-injected with the axis-inducing RNA. Expression from this reporter plasmid relies on an RA responsive element (RARE), which in turn depends on the availability of the RA ligand, the retinoic acid nuclear receptors, and their cofactors, i.e., an active RA signaling pathway. During gastrula (st. 10.25) and early neurula (st. 14/15) expression of the RAREZ reporter plasmid was detected irrespective of the mode of secondary axis induction employed (Supplementary Figure S1). During early gastrula, the induced secondary organizers exhibited RAREZ activity in about 76% of the embryos (Supplementary Figure S1C). This observation shows that although secondary dorsal lips, organizers, are induced, not all of them manage to activate the RAREZ reporter. It is important to note that activity of this reporter plasmid is totally dependent on the local biosynthesis of RA and expression of the RAR and RXR nuclear receptors. These results show that the induced secondary organizer also exhibits active RA signaling, although a more effective and efficient induction might be needed to activate a detectable RA signal trace.

As induced secondary organizers exhibit active RA signaling, we studied the requirement for this signal in the head organizer activity by studying the effect of reduced RA signaling on head development in secondary axis head induction. Ventral *wnt8a* mRNA injection was selected for axis induction as a large percentage of the secondary axes form anterior head structures (Sokol et al., 1991). Co-injection of *cyp26a1* mRNA together with the *wnt8a* RNA was used to reduce, in a localized manner, the RA level in the induced secondary axes. Embryos were processed for *in situ* hybridization with *ncam1* as a marker of the neural plate to score the secondary trunk, while anterior head formation was determined using *pax6* as an eye marker (Figures 1E–G). The efficiency of secondary axis induction was not significantly affected by the reduction in RA signaling compared to control embryos (61.3%, *n* = 856 and 55.5%, *n* = 1,450, respectively; Figure 1H). Analysis of the presence of anterior head structures morphologically and by marker gene expression showed that in the control group, 43.8% of the secondary axes had anterior head structures (Figures 1F,H). In contrast, RA knock-down reduced the proportion of secondary axes containing anterior head structures to only 27.9% of all the injected embryos, representing a reduction of 36.3% in full secondary axes (Figures 1G,H). These results show that RA is also required for the head organizer activity in induced secondary axes, similar to the endogenous organizer.

Additional support for the requirement for RA during head formation was obtained using additional means of secondary axis induction and RA signaling inhibition. Embryos were injected ventrally with *wnt8a* mRNA and subsequently treated with the RALDH inhibitor, DEAB. The DEAB treatment reduced the efficiency of head formation in the secondary axes by 59.6% (*n* = 328; Figure 1H), further supporting that RA is required for head formation. In addition, we induced head-containing secondary axes by simultaneous inhibition of BMP and Wnt signaling by co-injection of RNA encoding the dominant-negative BMP receptor, tALK3, and the Wnt antagonist, DKK1 (Glinka et al., 1998). Similar to the previous combined treatments, DEAB-mediated inhibition of RA biosynthesis resulted in a 41.1% decrease in head formation in the induced secondary axes (*n* = 344; Figure 1H). We conclude that RA signaling is required for efficient anterior head structure development both in the endogenous and induced organizers.

### Retinoic Acid is Required for Normal Head Formation During Early Gastrula

**FIGURE 2:**
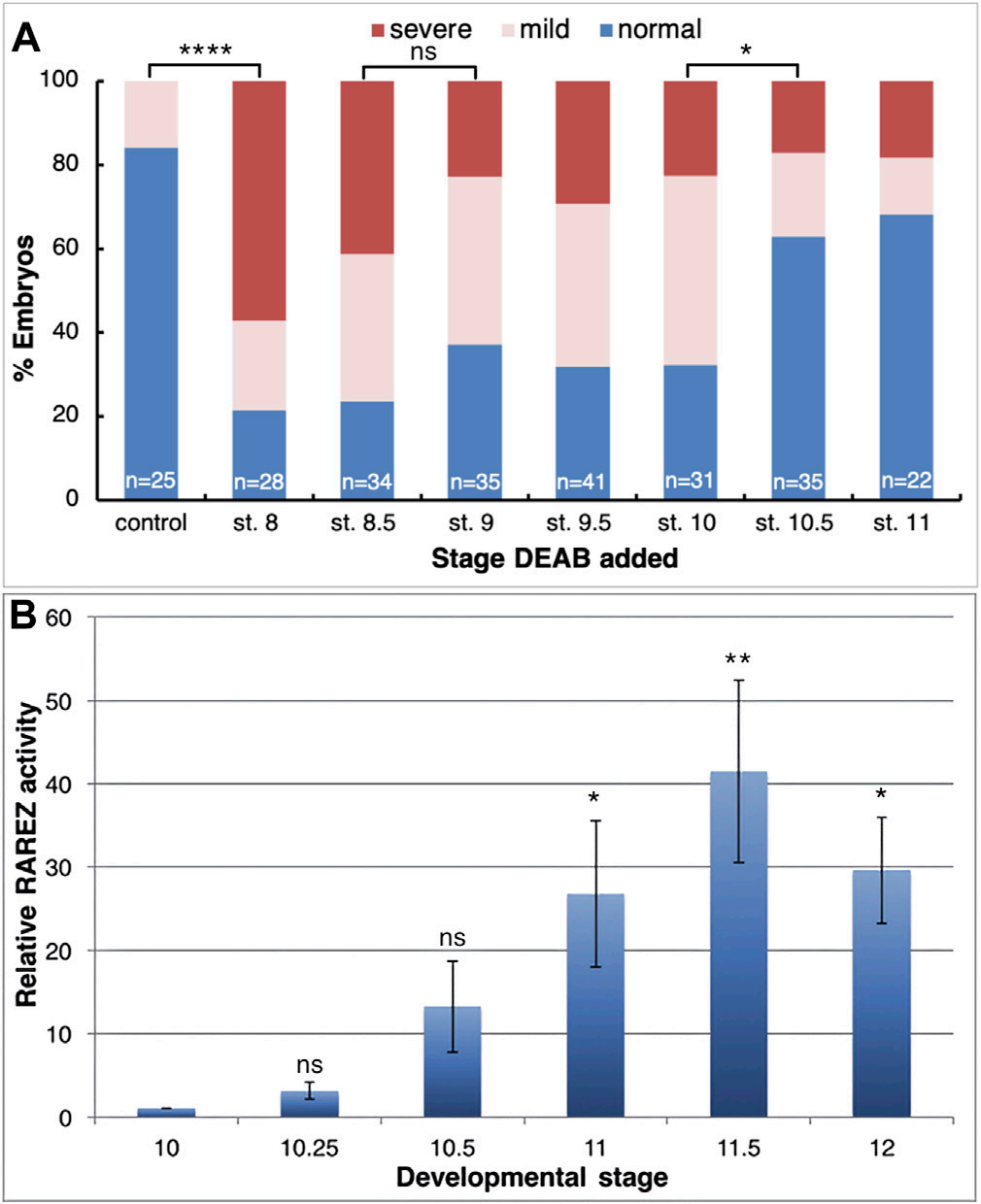
RA function is required during the early gastrula stages for normal anterior head development. **(A)** Embryos were treated with DEAB starting at different developmental stages and analyzed for the effect on head development during tailbud stages (st. 32). **(B)** Untreated embryos were injected with the RA reporter plasmid, RAREZ. At different gastrula stages, groups of embryos (5) were collected and processed for chemiluminescent analysis of the ß-galactosidase activity. Samples were normalized to the st. 10 sample. *, *p* < 0.05 **, *p* < 0.01; ****, *p* < 0.0001; ns, not significant.

To map the developmental window when RA signaling is required for normal head development, we inhibited RA biosynthesis by initiating the DEAB treatment at different developmental stages and analysis of the resulting head malformations at st. 32 (Figures 1A–C). Inhibition of RA synthesis from mid to late blastula stages (st. 8-9) resulted in 62–78% of the embryos developing microcephaly (Figure 2A). In the st. 8 sample, most embryos (57%) developed severe head malformations and 21% of them had mild head defects (Figure 2A). DEAB treatment from early gastrula (st. 10) resulted in a similar frequency of affected embryos (Figure 2A). Inhibition of RA biosynthesis from mid gastrula (st. 10.5) onwards showed a significant decrease in the induction of microcephaly, identifying a shift in the requirement for RA in the head organizer; only 37% of the embryos exhibit some form of microcephaly, 20% mild and 17% severe (Figure 2A). These results show a requirement for RA signaling during late blastula-early gastrula for normal head development.

RA and its biosynthetic intermediates have been detected in the gastrula organizer in vertebrate embryos (Chen et al., 1992; Hogan et al., 1992; Creech Kraft et al., 1994; Chen et al., 1994; Kraft et al., 1994; Niederreither et al., 1997; Chen et al., 2001; Yelin et al., 2005), suggesting an active role for this signaling pathway in this central embryonic regulatory structure. It is generally accepted that the appearance of the retinaldehyde dehydrogenase activity with the onset of *aldh1a2* (*raldh2*) expression marks the completion of the biosynthetic pathway and the onset of RA signaling (Ang and Duester, 1999; Niederreither et al., 1999; Chen et al., 2001; Shabtai et al., 2018). To obtain a better picture of the onset of RA signaling in the embryo, we took advantage of the RA reporter plasmid, RAREZ (Rossant et al., 1991), and determined the kinetics of increase in ß-galactosidase activity by chemiluminescence for maximal sensitivity. Embryos injected radially with the RAREZ plasmid (Yelin et al., 2005) were analyzed at different developmental stages from the midblastula transition (MBT; st. 8.5) to late gastrula (st. 12) (Figure 2B). This analysis shows that the RA reporter plasmid becomes active at the onset of gastrulation (st. 10-10.25) and its activity increases towards mid/late gastrula stages (st. 11.5-12). This temporal pattern of RAREZ activity places the onset of RA signaling around the beginning of gastrulation and, from then on, it increases towards late gastrula overlapping with the expression of *aldh1a2* (Chen et al., 2001; Shabtai et al., 2018) and the proposed activity of the head organizer and the transition to trunk organizer (Niehrs, 2004).

### Retinoic Acid is Required for Normal Gene Expression in the Head Organizer

**FIGURE 3:**
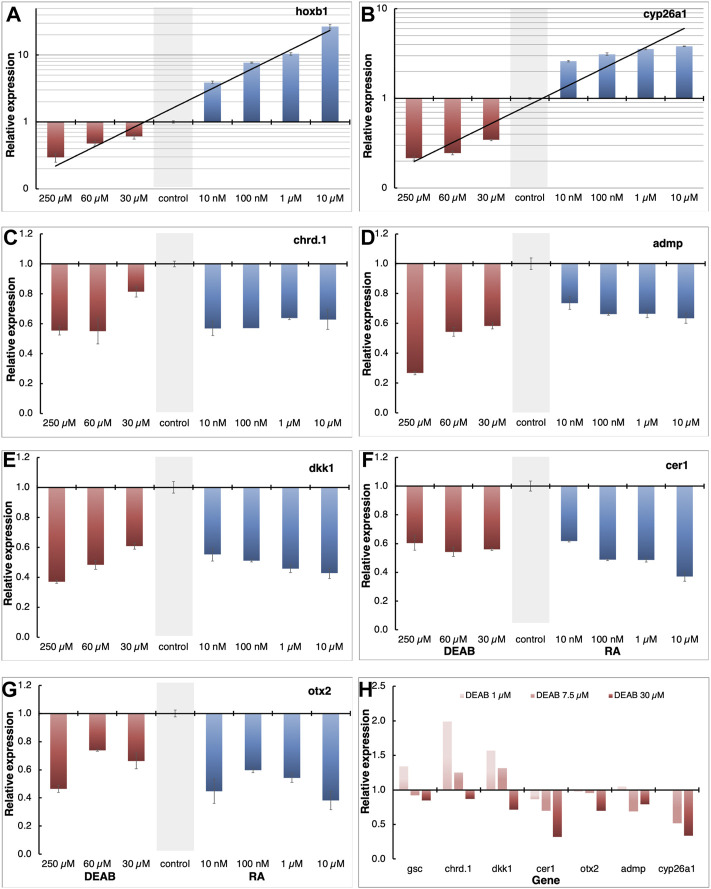
Positive and negative regulation of organizer genes by RA. **(A–G)** Embryos were treated with increasing concentrations of atRA (10 nM–10 μM) or DEAB (30–250 μM) from late blastula to early gastrula. Expression changes of *hoxb1* **(A)**, *cyp26a1* **(B)**, *chrd.1* **(C)**, *admp* **(D)**, *dkk1* **(E)**, *cer1* **(F)**, and *otx2* **(G)** were studied by qPCR. Samples were normalized to control expression levels (gray bar). **(H)** Fine titration of RA biosynthesis inhibition using DEAB (1–30 μM). Expression changes were determined for *gsc*, *cyp26a1*, *chrd.1*, *admp*, *dkk1*, *cer1*, and *otx2* by qPCR.

Using transgenic embryos, we previously showed that the RA pathway is active during early/mid gastrula mainly in the organizer and subsequently along the dorsal midline (Yelin et al., 2005). The early requirement for RA signaling for normal head development led us to study the role of RA within the organizer at a stage when both RA producing enzymes, ALDH1A2 and ALDH1A3 are expressed in this embryonic region. We manipulated the endogenous level of RA in the embryo and determined the effect of such manipulations on organizer-specific gene expression and, in particular, genes important for the head organizer activity. To decrease or increase the RA signaling levels, embryos were treated with increasing concentrations of DEAB (30-250 μM), or all*-trans* retinoic acid (atRA; 10 nM–10 μM) respectively, and incubated to early gastrula stages (st. 10.25) for expression analysis. To support the generation of different RA signaling levels we monitored the expression of the RA-regulated genes, *hoxb1* and *cyp26a1* by qPCR. This analysis revealed that our manipulations efficiently created an RA activity gradient ranging from a strong knockdown to gain-of-function (Figures 3A,B). Under these conditions, we studied the effect of RA manipulation on the expression of the organizer-specific genes; *cer1*, *admp*, *dkk1*, *chrd.1*, and *otx2*. The decrease in RA levels resulted in the downregulation of all the genes studied (Figures 3C–G). The downregulation ranged from about 40 to 60% from normal expression levels, suggesting that RA is a required signal for the normal expression of these organizer genes. Surprisingly, increasing the RA levels also reduced the expression of all genes studied by about 40–60% (Figures 3C–G). These results suggest that the embryo has close to optimal amounts of RA in the early gastrula organizer and any deviation results in reduced expression of the organizer genes studied.

To obtain support of whether the organizer normally contains almost optimal levels of RA to control the expression level of genes like *gsc, cer1*, *admp*, *dkk1*, *chrd.1*, and *otx2*, we performed a fine titration of the inhibition of RA biosynthesis using DEAB (Figure 3H). For *gsc*, *chrd.1*, and *dkk1*, the lowest amount of DEAB used (1 μM) resulted in upregulation of their expression, whereas higher concentrations had either no effect or had an opposite effect and downregulated their expression (Figure 3H). For *cer1*, *otx2*, *admp*, and *cyp26a1*, low DEAB concentrations had no effect, but higher concentrations induced lower expression levels (Figure 3H). These results show that RA has a complex gene regulatory role within the organizer, both positively and negatively controlling the levels of gene expression in a concentration and threshold-dependent manner to fine-tune the expression of the organizer genes.

### Expression of aldh1a2, and aldh1a3 During Gastrula Stages

**FIGURE 4:**
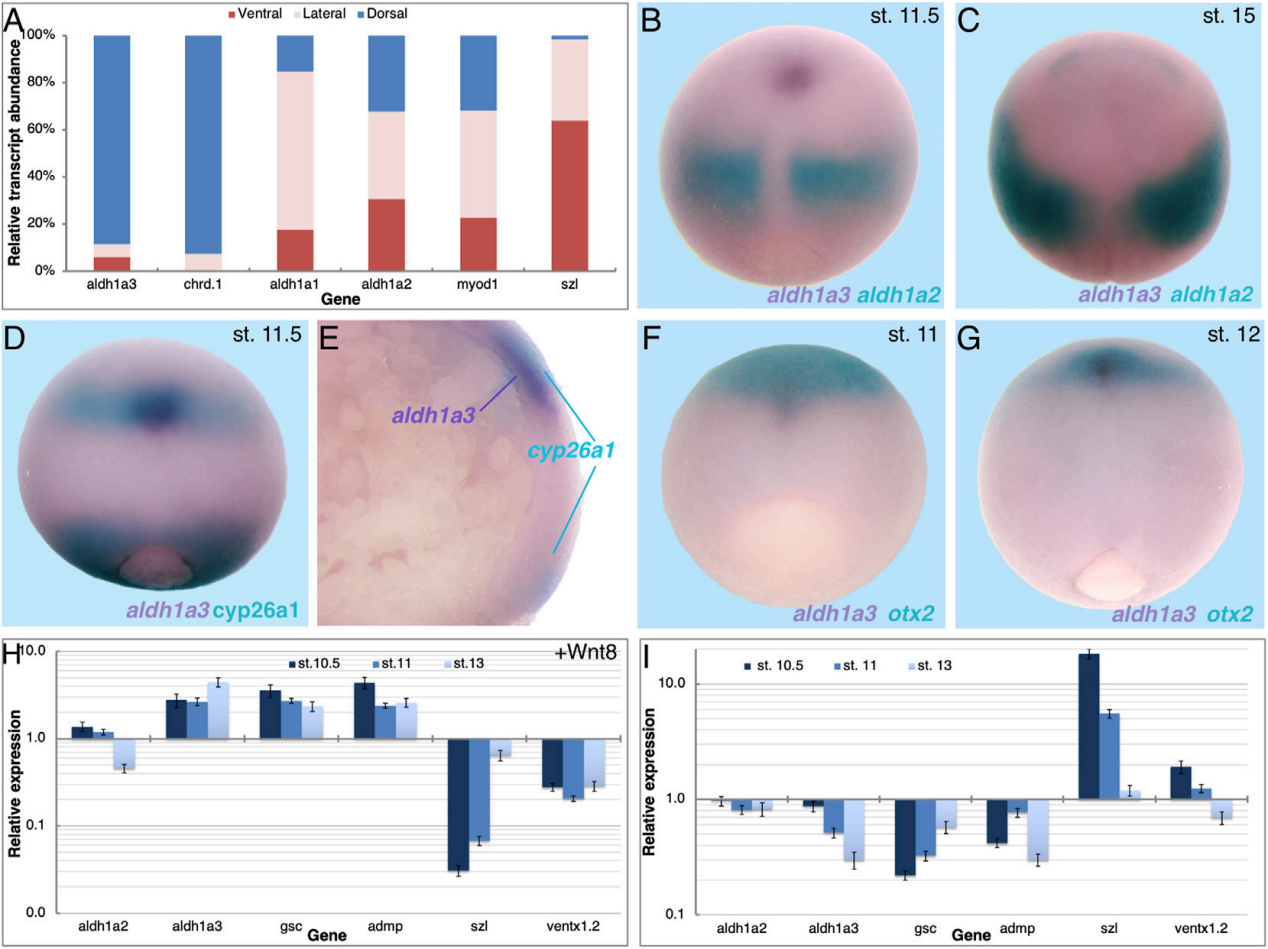
Expression of *aldh1a3* in the migrating LEM/PCM cells. **(A)** Mid-gastrula embryos (st. 11) were dissected into dorsal, lateral, and ventral regions, and RNA was extracted from each region. The relative abundance of *aldh1a1*, *aldh1a2*, and *aldh1a3* was studied by qPCR. The accuracy of the dissections was determined by qPCR of *chrd.1*, *myod1*, and *szl* as dorsal, lateral, and ventral markers, respectively. In comparison to the *szl* transcript distribution, all other genes had a significantly different distribution, *p* < 0.0001 using the Fisher exact probability test. Spatial expression pattern comparison between *aldh1a3* and *aldh1a2* **(B,C)**, *aldh1a3* and *cyp26a1* **(D,E)**, and *aldh1a3* and *otx2* **(F,G)**. **(B–D,F,G)** dorsal view, anterior to the top. **(H,I)** Embryos were injected with mRNA encoding WNT8a **(H)** or BMP4 **(I)** and samples were collected during early and mid-gastrula, and early neurula stages (st. 10.5, 11, and 13). qPCR analysis was performed for *aldh1a2* and *aldh1a3*, the dorsal markers *gsc* and *admp*, and the ventral genes *szl* and *ventx1.2*. Relative expression was normalized to levels in control embryos. Groups of injected embryos were incubated to tailbud stages to determine their dorsoanterior index (DAI) (Kao and Elinson, 1988). *wnt8a*, DAI = 5.67; *bmp4*, DAI = 3.64.

The results show that RA signaling is required during late blastula or early gastrula for normal organizer-specific gene expression and this early function contributes to the activity of the head organizer. We previously described the temporal pattern of expression of the three retinaldehyde dehydrogenase-encoding genes, *aldh1a1*, *aldh1a2*, and *aldh1a3* (*raldh1*, *raldh2*, and *raldh3*, respectively) in early *Xenopus* embryos and their relative transcript abundance (Shabtai et al., 2018; Parihar et al., 2021). The results showed that all three genes exhibit similar temporal expression patterns and are upregulated above background levels with the onset of gastrulation, but *aldh1a2* is the most abundant transcript. Since transcripts of *aldh1a2* and *aldh1a3* have been detected in the embryonic organizer (Chen et al., 2001; Lupo et al., 2005) we compared their spatial expression patterns by qPCR and double whole-mount *in situ* hybridization (dWISH) later during gastrulation. The relative localization of the *aldh1a1*, *aldh1a2,* and *aldh1a3* transcripts was determined by dissecting dorsal, lateral, and ventral marginal zones (DMZ, LMZ, and VMZ, respectively) from embryos during mid-gastrula (st. 11). The RNA from these regions was analyzed by qPCR to determine the relative transcript distribution (Figure 4A). Although expressed at very low levels (Shabtai et al., 2018), most of the *aldh1a1* transcripts are localized in the LMZ explants (Figure 4A). With this type of analysis, *aldh1a2* expression appears ubiquitous with similar abundance in all three regions in agreement with its wide expression domain during mid/late gastrula stages as observed by WISH (Figures 4B,C, turquoise). Expression of *aldh1a3* is mostly localized to the DMZ with almost no transcripts in other embryonic regions (Figure 4A). This *aldh1a3* transcript distribution is in agreement with the expression being restricted to the organizer and to cells along the dorsal midline, suggesting that this gene might be involved in the head-promoting role of RA. The accuracy of the embryonic dissections was corroborated by analyzing the expression of *chrd.1* as a dorsal marker, *myod1* as a lateral marker, and *szl* as a ventral marker (Figure 4A).

To better understand the pattern of *aldh1a3* expression, we studied its spatial expression in parallel to *aldh1a2*, *cyp26a1*, and *otx2*. Comparative analysis of the *aldh1a2* and *aldh1a3* expression patterns clearly shows that by mid/late gastrula (st. 11.5) both genes are expressed in non-overlapping domains (Figure 4B). While the *aldh1a2* expression remains posterior, close to the blastopore, *aldh1a3* expression is restricted to a small cluster of cells, probably representing the migrating LEM/PCM (Figure 4B). By early neurula stages (st. 15) *aldh1a3* expression becomes undetectable and only the expression of *aldh1a2* in the prospective trunk is observed (Figure 4C). The rostral localization of *aldh1a3* expression during late gastrula prompted us to look at its position relative to other genes expressed in the same region. First, we analyzed the spatial localization relative to *cyp26a1*, another member of the RA metabolic network known to be expressed in the neuroectoderm of the prospective forebrain region (Hollemann et al., 1998; de Roos et al., 1999). The dWISH results show that the *aldh1a3* expressing cells (purple) appear to overlap with the cranial domain of *cyp26a1* expression (turquoise; Figure 4D). To better understand this apparent overlap between the *aldh1a3* and *cyp26a1* expression domains in the rostral region, embryos were bisected sagittally for analysis. While the *cyp26a1* expression localizes to more superficial cells in the rostral as well as the blastopore expression domain, the *aldh1a3* expressing cells localize just below the rostral *cyp26a1* positive cells (Figure 4E). Although we cannot rule out a slight overlap in the future head domain, *cyp26a1* is expressed in the ectodermal cells whereas *aldh1a3* is expressed in the migrating mesendodermal cells. To better understand the relative position of the *aldh1a3*-positive cells within the rostral domain we compared it to the *otx2* expression domain, another early anterior head marker (Blitz and Cho, 1995; Pannese et al., 1995). Analysis of the overlap shows a small group of *aldh1a3* positive cells (purple) migrating rostrally towards the prospective midbrain/forebrain domain marked by *otx2* expression during mid gastrula (st. 11) (turquoise; Figure 4F). During late gastrula (st. 12), the small cluster of *aldh1a3* positive cells can be detected beneath a larger *otx2* expressing domain (Figure 4G). Also, in this case, the *aldh1a3* expressing cells localize ventrally to the *otx2* expression domain. These results support the conclusion that *aldh1a3* is expressed in the anterior mesendodermal, LEM/PCM cells.

The enzymatic function of *aldh1a3* as a producer of RA places a dynamic, second RA signaling center in the dorsal region of the gastrula embryo. The expression pattern of *aldh1a3* during gastrula and early neurula is characteristic of organizer genes that continue to have dorsal midline expression. To further study the dorsal identity of *aldh1a3*-expressing cells, embryos were dorsalized by promoting Wnt/ß-catenin signaling, or ventralized by increasing the BMP signal. Embryos were injected with *wnt8a* or *bmp4* mRNA to induce dorsalization and ventralization, respectively, and samples were collected during early/mid gastrula (st. 10.5), mid gastrula (st. 11), and early neurula (st. 13) to account for the dynamic nature of the expression patterns. To verify the efficacy of the RNA injections, we studied the responses of *gsc* and *admp* as dorsal genes and *szl* and *ventx1.2* as ventral genes by qPCR. In agreement with their dorsal expression, *gsc* and *admp* were upregulated by *wnt8a* RNA injection dorsalization and downregulated by *bmp4* ventralization (Figures 4H,I). *szl* and *ventx1.2*, on the other hand, exhibited the expected opposite responses as ventral targets of BMP4 signaling, downregulation by *wnt8a*, and upregulation by *bmp4* overexpression (Figures 4H,I). At all stages studied, *aldh1a3* was upregulated by *wnt8a* overexpression and downregulated by *bmp4*, similar t*o gsc* and *admp* (Figures 4H,I). These results show that *aldh1a3* responds like a typical organizer and dorsal midline gene to the manipulation of dorsal-ventral patterning. In contrast, from stage 10.5, the *aldh1a2* domain of expression expands laterally and its transcripts are eliminated from the organizer or midline region (Figures 4A–C) (Chen et al., 2001). In agreement, activation of the early Wnt/ß-catenin pathway has a very slight upregulatory effect, and during later stages, it weakly represses *aldh1a2* expression. The changes induced by Wnt/ß-catenin activation emphasize the dynamic changes in *aldh1a2* expression with the progression of gastrulation (Figure 4H) (Chen et al., 2001). Manipulation of the BMP signal had no effect on the *aldh1a2* expression (Figure 4I).

### ALDH1A3 Produces Retinoic Acid Required for Head Formation

Characterization of the *aldh1a3* and *aldh1a2* expression patterns showed that while *aldh1a2* expression begins in the organizer and then expands and shifts laterally, *aldh1a3* expression begins in the organizer and until late gastrula remains restricted to the dorsal midline, rostrally migrating LEM/PCM cells (Figures 4B,C). From the shared expression patterns in the gastrula organizer and the timing of the RA signal we were unable to identify whether one of these enzymes has a more prominent role in normal head development. To functionally determine which RA-producing enzyme contributes to head formation, we designed antisense morpholino oligonucleotides (MO) targeting either *aldh1a3* or *aldh1a2* (R3MO or R2MO, respectively). To determine the efficiency and specificity of R2MO and R3MO we constructed GFP variants containing the *aldh1a3* and *aldh1a2* MO target sequences (R3GFP or R2GFP, respectively). RNAs encoding the GFP variants were co-injected with the R3MO, R2MO, or control MO (coMO) into *Xenopus* embryos (Supplementary Figure S2). Only when R2GFP was co-injected with the R2MO or R3GFP was co-injected with the R3MO, was the GFP fluorescence strongly reduced (Supplementary Figures S2D,I). Co-injection with the control MO had no effect on the fluorescence intensity of the GFP variants (Supplementary Figures S2B,C,G,H). These results demonstrate that R2MO and R3MO are efficient tools for the knock-down of their respective proteins.

**FIGURE 5:**
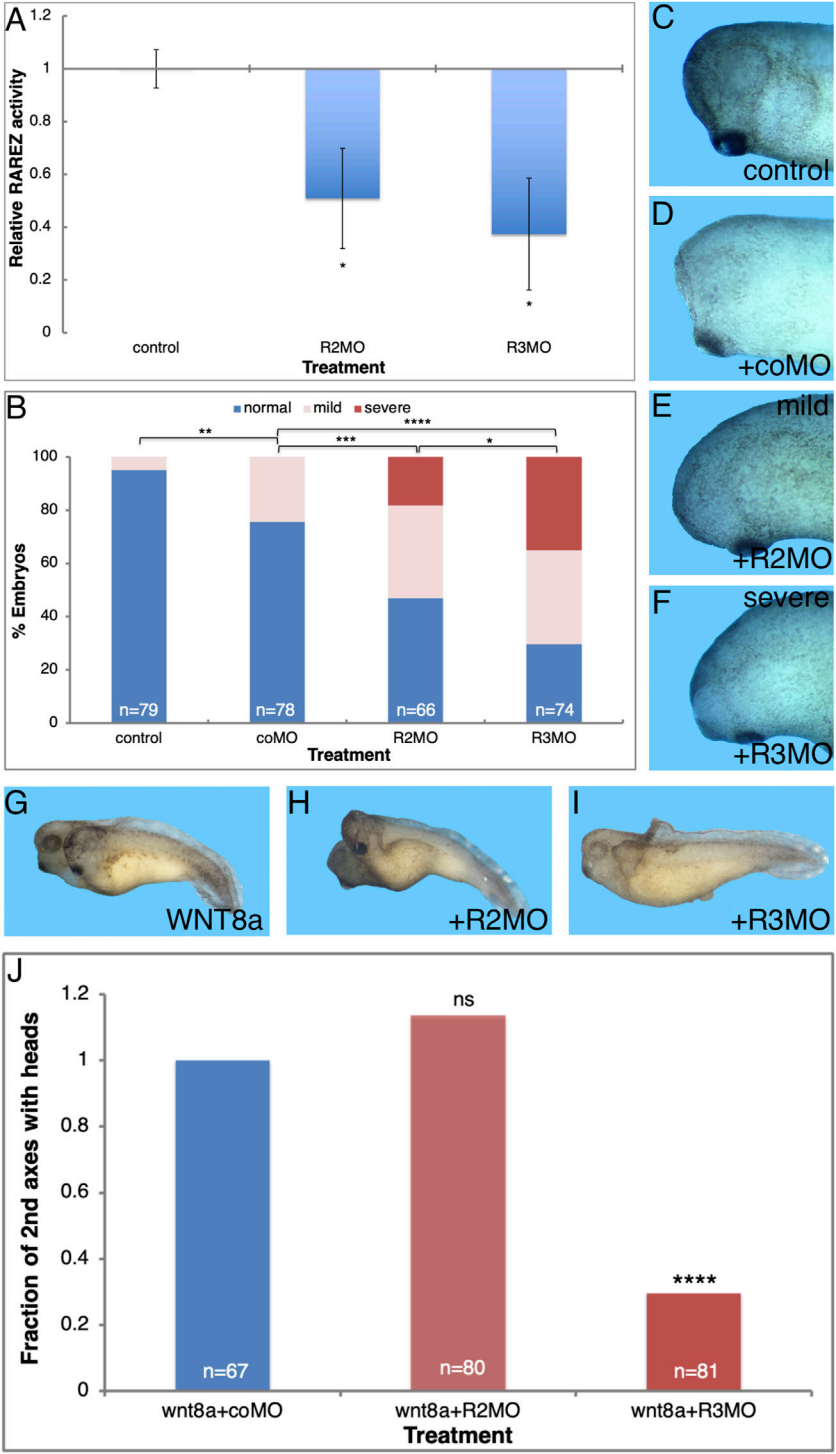
ALDH1A3 is necessary for normal head development. Embryos were injected with the R2MO or R3MO to reduce the activity of ALDH1A2 or ALDH1A3, respectively. **(A)** Analysis of the effect on the RA signaling level by co-injection of the RA reporter plasmid and chemiluminescent analysis of the ß-galactosidase activity. **(B–F)** Embryos injected dorsally with the R2MO, R3MO, or coMO to induce ALDH1A2 or ALDH1A3 knockdown in the Spemann-Mangold organizer. Embryos were sensitized for changes in RA levels by co-injection of low, non-teratogenic, amounts of *cyp26a1* RNA. The extent of microcephaly induction was quantitated **(B)**. Examples of head development for control uninjected **(C)**, coMO **(D)**, R2MO **(E)**, and R3MO **(F)** injected embryos are shown. **(G–J)** Analysis of head malformations in secondary axes induced by ventral injection of *wnt8a* RNA together with ALDH1A2 or ALDH1A3 knockdown. **(G)** Control embryo. **(H)** ALDH1A2 knockdown. **(I)** ALDH1A3 knockdown. **(J)** Quantitation of the effect of ALDH1A2 or ALDH1A3 knockdown on head development in the induced secondary axes. The overall number of embryos injected or manipulated is shown (*n* =). *, *p* < 0.05 **, *p* < 0.01; ***, *p* < 0.001 ****, *p* < 0.0001; ns, not significant.

Knockdown of the ALDH1A3 or ALDH1A2 enzymes is expected to reduce their activity and ultimately result in a reduction in RA signaling. For this reason, additional validation of the efficacy of the MOs directed against *aldh1a2* and *aldh1a3* (R2MO and R3MO) tested their effect on RA signaling levels. The effect of ALDH1A3 and ALDH1A2 knockdown on RA signaling was studied by co-injection with the RA reporter plasmid, RAREZ. Embryos injected with RAREZ and either R3MO or R2MO were collected at early/mid gastrula (st. 10.5) and the level of ß-galactosidase activity was determined using its chemiluminescent substrate (Figure 5A). This analysis showed that knockdown of either enzyme, ALDH1A3 or ALDH1A2, efficiently reduces the level of RA signaling by about 50–60% of control levels (Figure 5A). Thus, R3MO and R2MO efficiently hamper the production of RA, and both enzymes contribute to RA in the early gastrula embryo.

Our results of systemic RA reduction by CYP26A1 overexpression or the use of RA biosynthesis inhibition (DEAB) show that this signal is required for normal head development and its reduction results in microcephaly. Taking advantage of R2MO and R3MO we determined the relative contribution of ALDH1A3 and ALDH1A2 to normal head development. We reduced the expression of ALDH1A3 or ALDH1A2 by MO-mediated knockdown in the endogenous organizer by injecting embryos dorsally with R2MO, R3MO, or coMO, and the extent of induced microcephaly was quantitated (Figures 5B–F). The R2MO and R3MO efficiently induce knockdown of the endogenous activity, but we have shown that different embryo clutches respond differently to RA inhibition probably establishing a compensatory robustness response (Blum et al., 2015; Shukrun et al., 2019; Parihar et al., 2021). For these reasons, the experiment was performed in embryos sensitized for changes in RA levels by co-injection of low, non-teratogenic, amounts of *cyp26A1* RNA which weakly reduces the levels of RA (Figure 5D) to improve the effect of the MOs injected. The results show that under these conditions, ALDH1A3 knockdown induces severe microcephaly in a large proportion of embryos (35.1%; Figures 5B,F). By comparison, ALDH1A2 knockdown induces severe microcephaly in only 18.2% of the embryos (Figures 5B,E). The coMO had a weak effect on head morphology, inducing mild microcephaly in 24.6% of the embryos compared to 34.8 and 35.1% for R2MO and R3MO, respectively (Figures 5B,D). These results support the role of ALDH1A3 as providing the main retinaldehyde dehydrogenase activity producing the RA that is required for head formation, whereas ALDH1A2 appears to play a less prominent role in head development.

To corroborate the requirement for the function ALDH1A3 in normal head formation, we studied head-containing secondary axes induced by ventral injection of *wnt8a* mRNA. The role of ALDH1A3 or ALDH1A2 in head formation was studied by injection of the R2MO or R3MO together with the induction of the secondary axis (Figures 5G,H). ALDH1A3 knockdown dramatically reduced the efficiency of head formation in the induced secondary axes by 70% (Figures 5I,J); in contrast, ALDH1A2 knockdown did not affect the efficiency of head formation as compared to control *wnt8a* RNA injected embryos (Figures 5F,J). Neither morpholino significantly affects the efficiency of secondary axis induction (Figure 5J). These results show that ALDH1A3 has a strong effect on head development and appears to be a central RA producing enzyme required for this process.

To validate the MO results on head formation, we designed single guide RNAs (sg) to induce indels in either the *aldh1a2* or *aldh1a3* genes (sgR2 and sgR3, respectively) using the CRISPR/Cas9 approach (Tandon et al., 2017; Naert et al., 2020). The sgR2 and sgR3 RNAs were designed to target both homoeologs of either *aldh1a2* or *aldh1a3*, respectively (Table 1; Supplementary Figures S3A,B). One-cell embryos were injected with RNP complexes of either sgR2 or sgR3 RNA together with Cas9 protein to generate CRISPant embryos that were allowed to develop to early tailbud stages (st.32) for genomic DNA extraction. Sequencing of the genomic region targeted by the sgRNAs revealed clear disruption of the normal sequence (Supplementary Figures S3C–E). Decomposition analysis of the genomic sequences estimated a frameshift efficiency higher than 78% (Supplementary Figure S3C). The efficiency of the sgR2 and sgR3 allow us to perform gene editing experiments and analyze the injected founder (F0) individuals, CRISPants.

**FIGURE 6:**
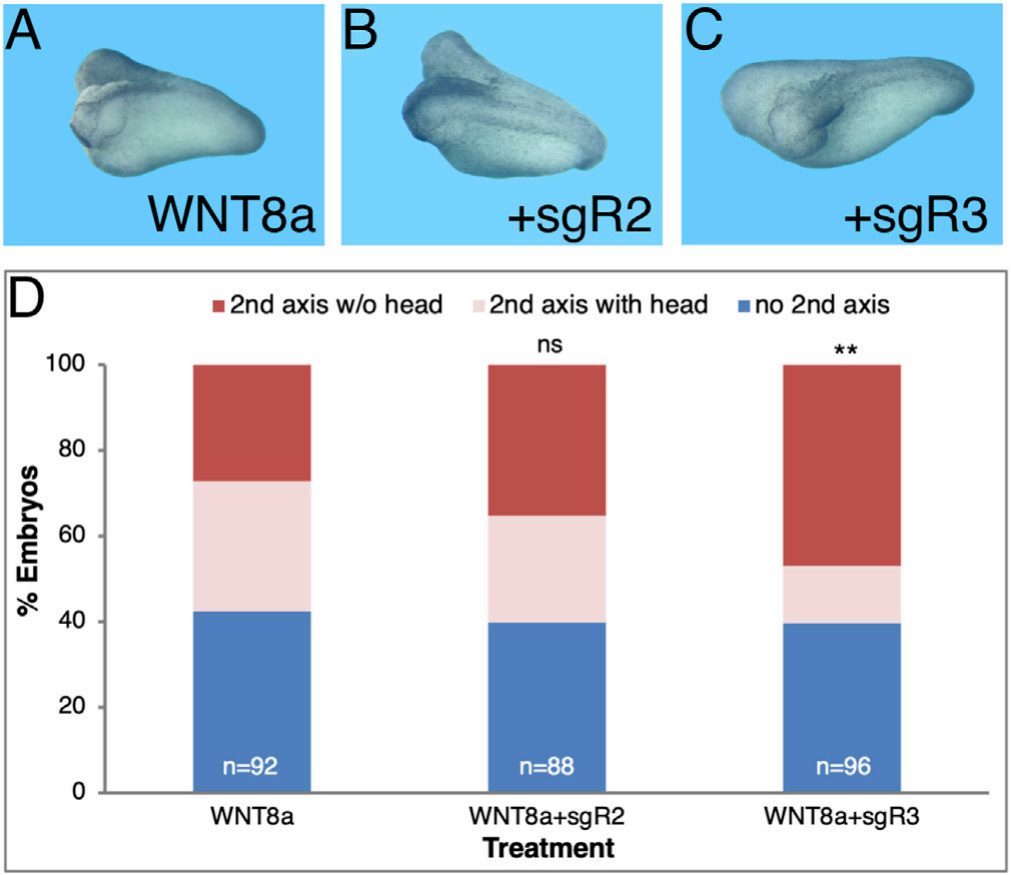
*aldh1a3* CRISPants exhibit enhanced head malformations. Secondary axes were induced by ventrally injecting *wnt8a* mRNA. Co-injection of sgR2 or sgR3 RNPs was performed to generate *aldh1a2* or *aldh1a3* CRISPant embryos, respectively. **(A)** Secondary axis induction efficiency in *wnt8a* RNA injected embryos along or in conjunction with *aldh1a2* or *aldh1a3* CRISPant induction. **(B)** Control embryo. **(C)** *aldh1a2* CRISPant embryo with two axes. **(D)** *aldh1a3* CRISPant twinned axis embryo. **(E)** Analysis of the effect of the *aldh1a2* and *aldh1a3* CRISPR/Cas9-mediated knockdown on head formation in the induced secondary axes. The overall number of embryos injected or manipulated is shown (*n* =). Percent embryos *wnt8a* mRNA injected, embryos with secondary axes without heads and with heads. **, *p* < 0.01; ns, not significant.

Taking advantage of the generation of CRISPant embryos, we performed the head formation inhibition assay in secondary axes by injecting CRISPR/Cas9 with either sgR2 or sgR3 (Figure 6). Embryos were injected ventrally with a combination of *wnt8a* RNA for secondary axis induction together with the sgRNA/Cas9 RNP complex. The sgRNA/Cas9 complex had no effect on the secondary axis induction efficiency which was around 60% of the injected embryos in control *wnt8a* only and sgR2 or sgR3 CRISPant embryos (Figures 6A,C). Indel induction in the *aldh1a3* CRISPants significantly increased the loss of secondary head formation efficiency (55.5% loss; Figures 6C,D). Knockdown of the ALDH1A2 activity had a slight (17.9%) and not significant effect on head formation in the secondary axes (Figures 6B,D). These results confirm that loss-of-function the ALDH1A3 activity by CRISPR/Cas9 gene targeting is critical for normal head development.

**FIGURE 7:**
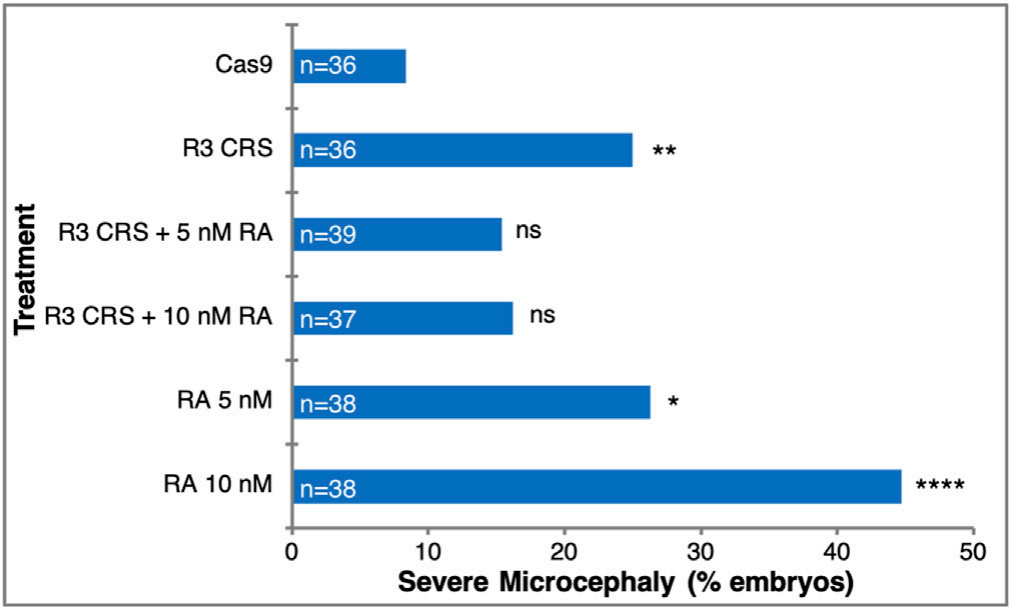
RA rescues the microcephaly induced by loss of ALDH1A3 activity. Microcephaly was induced in *Xenopus* embryos by targeting the *aldh1a3* gene with CRISPR/Cas9+sgR3. As controls, embryos were injected with Cas9 only. For rescue of the microcephalic phenotype, embryos were treated with 5 nM or 10 nM RA. The rescue efficiency was calculated by Chi-square, comparing each treatment to the Cas9 control microcephaly level. *, *p* < 0.05; **, *p* < 0.01; ****, *p* < 0.0001; ns, not significant.

The knockdown experiments suggest a novel function for RA signaling during gastrulation by providing a required signal for normal head development. To further support this RA requirement in head formation we performed rescue experiments. Microcephaly was induced by generating *aldh1a3* CRISPant embryos and the head malformations were rescued by the addition of low amounts of RA (5 nM or 10 nM) (Figure 7). Whereas, among Cas9 injected control embryos only 8.4% developed severe microcephaly, targeting the *aldh1a3* gene with sgR3 resulted in a significant three-fold increase (25%) of embryos with severe microcephaly (Figure 7). The addition of low amounts of RA to the *aldh1a3* CRISPant embryos reduced the extent of severe microcephaly by about a third (to 15.4–16.2%). It is important to note that low amounts of RA alone induced severe microcephaly (26.3% for 5 nM and 44.7% for 10 nM) through inhibition of forebrain fates. These results show that RA supplementation of *aldh1a3* CRISPant embryos partially rescues the microcephaly induced by the loss of the RA producing enzyme.

### Retinoic Acid Regulatory Functions During Early Head Formation

**FIGURE 8:**
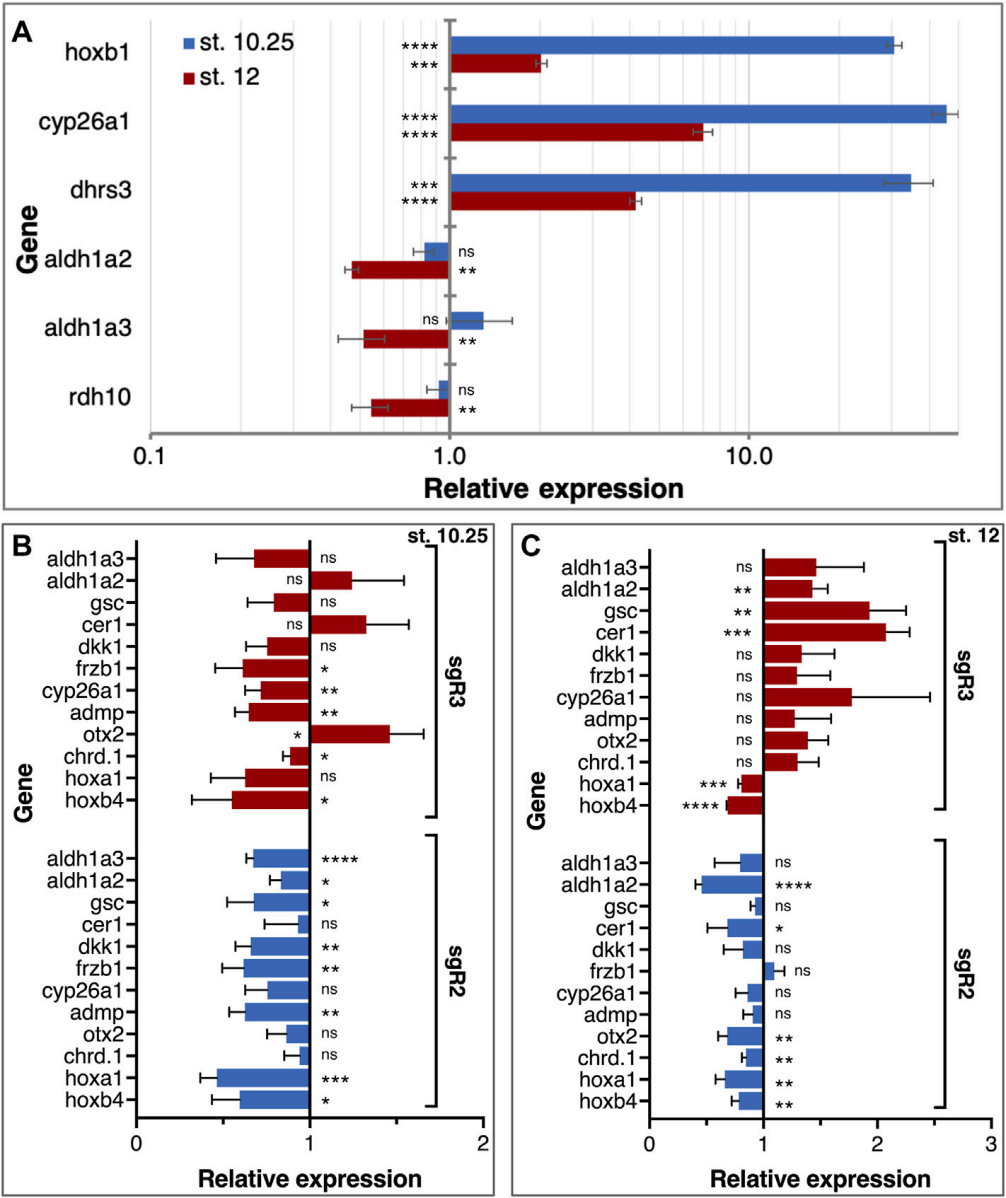
RA autoregulation and control of LEM/PCM gene expression. **(A)** Embryos were treated with RA (100 nM) during late blastula stages and RNA samples were collected during early (st. 10.25) and late (st. 12) gastrula stages. The effect of the manipulation on RA network gene expression (*dhrs3*, *cyp26a1*, *aldh1a2*, *aldh1a3*, and *rdh10*) was determined by qPCR. The expression of *hoxb1* was studied to monitor the changes in RA level. Samples were normalized to control expression level at each stage. **(B,C)** The expression of organizer genes linked to head formation (*gsc*, *cer1*, *dkk1*, *frzb1*, *admp*, *otx2*, and *chrd.1*) was analyzed in *aldh1a2* and *aldh1a3* CRISPants. The RA-regulated genes, *cyp26a1*, *hoxa1*, *hoxb4*, *aldh1a2*, and *aldh1a3*, were studied in parallel. The gene expression analysis was performed at st. 10.25 **(B)** and st. 12 **(C)**. Relative expression was normalized to control expression levels at each stage. *, *p* < 0.05; **, *p* < 0.01; ***, *p* < 0.001; ****, *p* < 0.0001; ns, not significant.

RA is well known to regulate the genes that affect its level. To better characterize the molecular, gene-regulatory role of RA signaling in the head organizer, we studied the effect of RA addition on the expression of *aldh1a2* and *aldh1a3* and a number of head organizer genes. The initial activation of *aldh1a2* expression is probably RA-independent but soon thereafter the RA self-regulation might contribute to the expression of RA network genes including *aldh1a2* and *aldh1a3*. To determine the responsiveness to increased levels of RA of gastrula expressed RA metabolic genes, we treated embryos with 100 nM RA from late blastula to early/mid (st. 10.25) and late gastrula stages (st. 12). Analysis of *hoxb1* expression revealed the expected RA-dependent upregulation at both gastrula stages supporting the efficiency of this treatment (Figure 8A). Similarly, the expression of genes important for attenuating RA signaling, *dhrs3*, and *cyp26a1*, was upregulated relative to controls in agreement with their enzymatic role (Figure 8A). In contrast, genes encoding RA biosynthetic enzymes, *aldh1a2*, *aldh1a3*, and *rdh10* exhibit weak, not significant responses to the RA increase during early gastrula stages (Figure 8A); *aldh1a2* and *rdh10* exhibit slight downregulation, whereas *aldh1a3* exhibits weak upregulation (Figure 8A). During late gastrula, however, all three RA biosynthetic genes exhibit a more robust and significant, RA-mediated downregulation (Figure 8A). These results indicate the very early establishment of RA self-regulatory network gene responses. While increased RA levels upregulate genes encoding enzymes that suppress the levels of RA from early gastrula, genes involved in the production of RA are downregulated only by late gastrula (Figure 8A).

To understand the contribution of the RA signaling centers during gastrulation to head development, we took advantage of the sgR2 and sgR3 gene-specific CRISPants and analyzed the effect on organizer genes previously shown to be involved in head development, i.e., head organizer genes. CRISPants were collected at early gastrula (st. 10.25) when the domains of *aldh1a2* and *aldh1a3* overlap, and during late gastrula when their domains are separate (Figure 4B). qPCR analysis of *gsc*, *cer1*, *dkk1*, *frzb1*, *admp*, *otx2*, and *chrd.1* was performed, in addition to a number of known RA-regulated genes: *cyp26a1*, *hoxa1*, *hoxb4*, *aldh1a2*, and *aldh1a3* (Figures 8B,C). During early gastrula, most genes studied exhibited some degree of downregulation in both *aldh1a2* and *aldh1a3* CRISPants (Figure 8B). This observation is in agreement with the required role of RA in the normal gene expression in the organizer and surrounding regions supporting the results of systemic RA manipulations (Figure 3). Interestingly, knockdown of either gene had similar effects suggesting that both enzymes contribute to the production of RA in the organizer. By late gastrula, however, the effect of the *aldh1a2* and *aldh1a3* CRISPants differs. While the gene expression changes to *aldh1a2* knockdown are very slight, in *aldh1a3* CRISPants most genes tested exhibited weak upregulation (Figure 8C). These results suggest that ALDH1A2 has a very limited effect on the expression of organizer genes that continue to be expressed in the LEM/PCM cells. In agreement with a role in head formation, ALDH1A3 knockdown affects most genes tested suggesting that in the LEM/PCM cells, RA modulates their expression.

## Discussion

### Retinoic Acid is Required for Head Formation

Induction of the anterior neuroectoderm including formation of the head is the focus of extensive study in developmental biology. In addition to the interest in the basic understanding of these processes, multiple human conditions arise from defects in these events including microcephaly and its accompanying cognitive disabilities. Numerous mutations or exposure to environmental factors can induce microcephaly in humans (Abuelo, 2007; Mochida, 2009; Dyment et al., 2013; Faheem et al., 2015; Duerinckx and Abramowicz, 2018). Alcohol (ethanol) exposure during pregnancy also induces microcephaly as part of the developmental malformations characteristic of FAS (Gautam et al., 2015; Popova et al., 2016; Del Campo and Jones, 2017; Jarmasz et al., 2017; Petrelli et al., 2019). Over the last years, evidence has accumulated showing that FAS induction by ethanol is mediated in part by a reduction in RA signaling (Kot-Leibovich and Fainsod, 2009; Muralidharan et al., 2015; Shabtai et al., 2018; Shukrun et al., 2019). This reduction in RA signaling and the resulting microcephaly in FAS suggested that RA signaling has a novel, and as yet unexplored function required for the normal formation of the head. In the present study we employed multiple assays to demonstrate the involvement of RA signaling in early head development. Irrespective of the assay used, RA knockdown resulted in a microcephalic phenotype similar to alcohol exposure (Nakatsuji, 1983; Shabtai et al., 2018; Shukrun et al., 2019).

It is commonly accepted that RA has an inhibitory function on the development of the rostral neuroectodermal domain by promoting hindbrain expansion and malformations (Durston et al., 1989; Sive et al., 1990; Koide et al., 2001; Ribes et al., 2007). The *Hox* genes are some of the earliest targets of RA signaling in the hindbrain and are thought to mediate this effect because many of the observed head malformations can be reproduced by their overexpression (Conlon and Rossant, 1992; Alexandre et al., 1996; Whiting, 1997; Zaffran et al., 2018). The prospective forebrain region is believed to be devoid of RA signaling during early embryogenesis based primarily on the expression of CYP26A1 in this head domain (Hollemann et al., 1998; Ribes et al., 2007; Tanibe et al., 2008; Nolte et al., 2019). Surprisingly, our analysis based on RA signaling knockdown using biosynthesis inhibitors or CYP26A1 overexpression resulted in the induction of microcephaly by reduced RA. This developmental malformation was obtained by targeting the embryonic organizer irrespective of whether the endogenous organizer was targeted or whether an ectopic, secondary organizer was experimentally induced and targeted. These results demonstrate that RA is required for normal head formation. Reduced RA signaling has previously been linked to forebrain malformations in mutants encoding components of the RA metabolic and signaling network like *aldh1a2*, *aldh1a3*, *rdh10*, and several *rar* genes (Lohnes et al., 1994; Mendelsohn et al., 1994; Dupé et al., 2003; Mic et al., 2004; Ribes et al., 2006; Molotkova et al., 2007; Sandell et al., 2007; Mark et al., 2009; Rhinn et al., 2011). Also, knockdown of RA network components like *sdr16c5* (*rdhe2*) or *rdh10* resulted in microcephalic phenotypes (Strate et al., 2009; Belyaeva et al., 2012). Vitamin A deficient quail embryos and *Xenopus* embryos treated with RA biosynthesis inhibitors also exhibit microcephaly (Halilagic et al., 2003; Halilagic et al., 2007; Kot-Leibovich and Fainsod, 2009; Shabtai et al., 2018). These studies probably describe several RA functions taking place at different developmental stages in different regions of the embryo (Petrelli et al., 2019), while the present study focuses on one of the earliest functions of RA signaling in the embryo.

### The Head-Promoting Activity of Retinoic Acid Localizes to the Organizer

Multiple studies have shown that RA is already present in the vertebrate embryonic organizer during early gastrula stages (Hogan et al., 1992; Creech Kraft et al., 1994; Kraft et al., 1994; Ulven et al., 2000). Functional RA signaling has been localized mainly to the embryonic organizer at similar stages (Rossant et al., 1991; Deltour et al., 1996; Yelin et al., 2005; Samarut et al., 2015). Our results using a reporter plasmid show that RA signaling becomes activated at the onset of gastrulation and continues to increase towards neurula stages. Activation of the RA pathway follows the temporal expression and transcript accumulation of *aldh1a2*, the retinaldehyde dehydrogenase activity required at those stages to complete the biosynthesis of RA (Ang and Duester, 1997; Niederreither et al., 1999; Grandel et al., 2002). Analysis of the temporal sensitivity window by RA biosynthesis inhibition at different developmental stages identified late blastula and the beginning of gastrulation as the window during which this signal is required for normal head development. These observations thus defined that the RA signal required for head formation initiates at around the onset of gastrulation and is localized to the gastrula organizer. In agreement, previous studies have shown that in *Xenopus* embryos, the LEM interacts with the prospective cranial neuroectoderm already during early gastrula stages (Koide et al., 2002).

Although RA accumulation and signaling in the organizer has been known for many years, the gene-regulatory function of this early RA signal has remained elusive. Taking advantage of the inhibition of RA biosynthesis or all*-trans* RA treatments we manipulated embryos creating samples that contain a gradient of RA concentrations above and below the normal endogenous amount. In these samples we studied the expression of organizer genes known to contribute to the formation of the head. The results showed that the unmanipulated embryo contains an almost optimal amount of RA and that experimentally induced small concentration changes in either direction results in reduced organizer-specific gene expression. Therefore, RA is normally required for the expression of all the organizer genes tested, but it also prevents the overexpression of these genes.

The observation that RA signaling in the early organizer is required for head formation raised a number of possibilities regarding the identity of the cells affected and the genetic network involved. The genes we analyzed in the RA manipulated embryos have all been shown to play an early role in the formation of the head (Matsuo et al., 1995; Dosch and Niehrs, 2000; Kuroda et al., 2004; Ishibashi et al., 2008; Tanaka et al., 2017). Together all this data would point to the subpopulation commonly termed the “head organizer” in *Xenopus* (Niehrs et al., 2001; Koide et al., 2002). The head, trunk and tail organizers are functional definitions of either subpopulations originating from the organizer or the inductive potential of the organizer at different times during embryogenesis (Kaneda and Motoki, 2012; Huang and Winklbauer, 2018). At the cellular level, one of the earliest cell populations invaginating and migrating rostrally in *Xenopus* embryos is the LEM that migrates cranially, and localizes below the prospective rostral neuroectoderm, a tissue they play a role in inducing.

### RALDH3 Produces the Retinoic Acid Needed for Head Formation

The results using inhibitors of RA biosynthesis (DEAB and citral) or degradation of the RA itself (CYP26A1) support a requirement for this signal during formation of the anterior head domain. To conclusively determine the involvement of RA in the early steps of head formation we set out to identify the source of this signal, i.e., the retinaldehyde dehydrogenase producing the RA for this activity. Two *aldh1a* genes are known to be expressed in the Spemann-Mangold organizer. *Aldh1a2* is the first retinaldehyde dehydrogenase expressed at the onset of gastrulation (Chen et al., 2001; Shabtai et al., 2018; Parihar et al., 2021). The appearance of this enzyme completes the biosynthesis of RA, making this pathway active apparently for the first time. This gene is initially expressed in the organizer, but by mid-gastrula the dorsal midline becomes devoid of transcripts and *aldh1a2* is expressed in more lateral regions (Chen et al., 2001). Our results show that *aldh1a3* is transcribed in a similar temporal pattern albeit at lower levels than *aldh1a2* (Shabtai et al., 2018), but with a different spatial pattern. *Aldh1a3* is co-expressed with *gsc* and *aldh1a2* in the early organizer and subsequently, remains co-expressed with *gsc* in the LEM/PCM as these cells migrate rostrally. The cells expressing *aldh1a3* within the prospective head domain appear to coincide axially with the *cyp26a1* and *otx2* expression domains but they are actually located beneath them. This pattern is in agreement with the PCM cells being a source of RA at these stages when the rostral neuroectoderm undergoes induction to form the anterior brain regions.

To characterize the function of ALDH1A3 we took advantage of a knockdown approach. We could show that reducing ALDH1A3 activity induces microcephaly and prevents head formation in a secondary axis induction assay. These results show that from its earliest expression, ALDH1A3 is present in the cells normally involved in the formation of the head. Thus, RA is required for the normal induction and formation of the head and a retinaldehyde dehydrogenase is expressed in the right cells at the right developmental stages. The source of RA for the head-forming activity is provided by the *aldh1a3*-expressing cells that induce the head. ALDH1A2 knockdown induced a weaker microcephaly suggesting that the ALDH1A3 activity might play a more central role in the induction and formation of the head and *aldh1a2* performs a very early function that can be partially compensated by *aldh1a3*.

### Positive and Negative Regulation of Rostral Head Domains by Retinoic Acid

**FIGURE 9:**
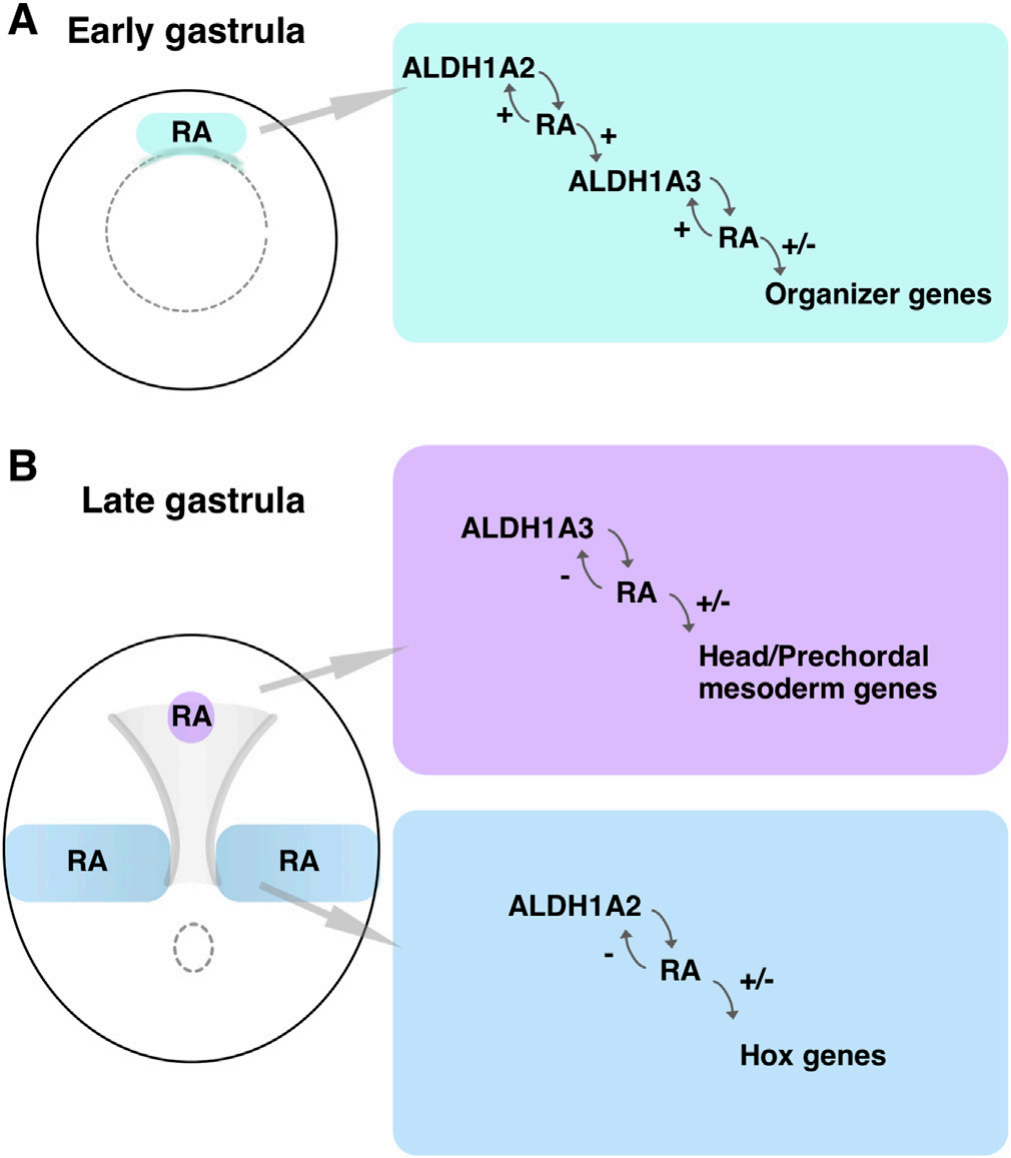
RA biosynthetic/signaling centers during gastrulation. **(A)** Schematic depiction of an early gastrula embryo where the domain of overlapping *aldh1a2* and *aldh1a3* expression in the Spemann-Mangold organizer is marked (green box). The RA producing and regulatory activities are summarized. **(B)** Schematic summary of the two RA biosynthetic/signaling centers during late gastrula. The domains of expression and activity of *aldh1a2* in the trunk (blue) and *aldh1a3* in the LEM/PCM (purple) are shown.

It is widely accepted that RA is a negative regulator of anterior brain regions based on extensive experimental evidence describing the transformation of anterior neural tissues to more posterior identities following RA treatment or mutation of genes involved in the attenuation of the RA signal (Durston et al., 1989; Sive et al., 1990; Hollemann et al., 1998; Koide et al., 2001; Ribes et al., 2007; Tanibe et al., 2008; Nolte et al., 2019). During early stages of brain development, the neuroectodermal region rostral to the midbrain-hindbrain boundary expresses CYP26A1 performing a protective role by hydroxylation and subsequent degradation of RA secreted from adjacent tissues (Koide et al., 2001; Weston et al., 2003; Tanibe et al., 2012; Zhong et al., 2019). Our results show that very early in gastrulation, RA signaling is also required for the development of a normal head. Soon after the onset of gastrulation, both *aldh1a2* and *aldh1a3* are expressed in the Spemann-Mangold organizer in *Xenopus* (Figure 9A). Similar expression patterns at comparative developmental stages have been described in other vertebrate embryos (Begemann et al., 2001; Blentic et al., 2003; Liang et al., 2008). Analysis of gene expression changes following RA manipulation and gene-specific knockdowns placed the RA produced by these enzymes as an important signal regulating multiple organizer genes (Figure 9A). Our results show that this early RA activity is required for normal head formation and possibly additional organizer functions. By early/mid gastrula the expression domains of *aldh1a2* and *aldh1a3* separate, establishing two RA biosynthetic/signaling centers (Figure 9B). The *aldh1a2* expression remains posterior, close to the blastopore, while the *aldh1a3*-expressing cells migrate cranially. The early cranially migrating cells, the LEM/PCM, will interact with the overlying ectoderm to induce the rostral neuroectoderm (Kaneda and Motoki, 2012; Huang and Winklbauer, 2018), and this interaction might take place very soon after the onset of migration (Koide et al., 2002; Lloret-Vilaspasa et al., 2010; Yanagi et al., 2015). These same cells express *aldh1a3,* whose knockdown results in microcephaly and abnormal expression of head organizer genes, further supporting an early role for RA signaling and ALDH1A3 in the formation of the head (Figure 9B).

A possible explanation for the apparent discrepancy between positive and negative regulation of head formation by RA could be a combination of timing and location. During early gastrula, we have previously described a delay in the invagination and migration of the LEM/PCM cells under reduced RA signaling conditions (Yelin et al., 2007; Yelin et al., 2005). In support of a role in the regulation of morphogenetic movements, vitamin A deficiency alters the extracellular matrix and the cellular activities dependent on it (Barber et al., 2014). On the other hand, the function of the RARs as protective during head formation was mainly studied during late gastrula/early neurula stages. The mid gastrula expression of the *rar* genes, *cyp26a1*, and the co-repressor genes in the ectoderm, localizes mainly to the prospective rostral neuroectoderm suggests an early protective function from neighboring RA source(s) (Hollemann et al., 1998; Koide et al., 2001). Analysis of the *raldh3* and *cyp26a1* transcripts in the prospective head region showed adjacent expression domains, which might be relevant to later functions of RA signaling in neuroectodermal differentiation. Then, reduced RA signaling could induce microcephaly by affecting the morphogenetic movements of the *aldh1a3*-expressing LEM/PCM cells out of the Spemann-Mangold organizer, or by directly affecting the inductive signals from these cells.

## Data Availability

The original contributions presented in the study are included in the article/Supplementary Material, further inquiries can be directed to the corresponding author.

## Abbreviations

ALDH, aldehyde dehydrogenase; EAE, embryonic alcohol exposure; FASD, Fetal Alcohol Spectrum Disorder; FAS, Fetal Alcohol Syndrome; LEM, leading edge mesendoderm; PCM, prechordal mesoderm; RA, retinoic acid; RAL, retinaldehyde; ROL, retinol; SDR, short-chain dehydrogenase/reductase.

## References

[B1] Abuelo D. (2007). Microcephaly Syndromes. Semin. Pediatr. Neurol. 14, 118–127. 10.1016/j.spen.2007.07.003 17980308

[B2] Alexandre D. Clarke J. D. Oxtoby E. Yan Y. L. Jowett T. Holder N. (1996). Ectopic Expression of Hoxa-1 in the Zebrafish Alters the Fate of the Mandibular Arch Neural Crest and Phenocopies a Retinoic Acid-Induced Phenotype. Development 122, 735–746. 10.1242/dev.122.3.735 8631251

[B3] Ang H. L. Duester G. (1997). Initiation of Retinoid Signaling in Primitive Streak Mouse Embryos: Spatiotemporal Expression Patterns of Receptors and Metabolic Enzymes for Ligand Synthesis. Dev. Dyn. 208, 536–543. 10.1002/(SICI)1097-0177(199704)208:4<536::AID-AJA9>3.0.CO;2-J 9097025

[B4] Ang H. L. Duester G. (1999). Stimulation of Premature Retinoic Acid Synthesis in Xenopus Embryos Following Premature Expression of Aldehyde Dehydrogenase ALDH1. Eur. J. Biochem. 260, 227–234. 10.1046/j.1432-1327.1999.00139.x 10091603

[B5] Barber T. Esteban-Pretel G. Marín M. Timoneda J. (2014). Vitamin a Deficiency and Alterations in the Extracellular Matrix. Nutrients 6, 4984–5017. 10.3390/nu6114984 25389900 PMC4245576

[B6] Begemann G. Schilling T. F. Rauch G.-J. Geisler R. Ingham P. W. (2001). The Zebrafish Neckless Mutation Reveals a Requirement for raldh2 in Mesodermal Signals that Pattern the Hindbrain. Development 128, 3081–3094. 10.1242/dev.128.16.3081 11688558

[B7] Belyaeva O. V. Lee S.-A. Adams M. K. Chang C. Kedishvili N. Y. (2012). Short Chain Dehydrogenase/reductase Rdhe2 is a Novel Retinol Dehydrogenase Essential for Frog Embryonic Development. J. Biol. Chem. 287, 9061–9071. 10.1074/jbc.M111.336727 22291023 PMC3308774

[B8] Blentic A. Gale E. Maden M. (2003). Retinoic Acid Signalling Centres in the Avian Embryo Identified by Sites of Expression of Synthesising and Catabolising Enzymes. Dev. Dyn. 227, 114–127. 10.1002/dvdy.10292 12701104

[B149] Blitz I. L. Cho K. W. (1995). Anterior neurectoderm is progressively induced during gastrulation: the role of the Xenopus homeobox gene orthodenticle. Development 121, 993–1004. 10.1242/dev.121.4.993 7743941

[B9] Blum M. De Robertis E. M. Wallingford J. B. Niehrs C. (2015). Morpholinos: Antisense and Sensibility. Develop. Cel 35, 145–149. 10.1016/j.devcel.2015.09.017 26506304

[B10] Brinkman E. K. Chen T. Amendola M. van Steensel B. (2014). Easy Quantitative Assessment of Genome Editing by Sequence Trace Decomposition. Nucleic Acids Res. 42, e168. 10.1093/nar/gku936 25300484 PMC4267669

[B11] Catharine Ross A. Zolfaghari R. (2011). Cytochrome P450s in the Regulation of Cellular Retinoic Acid Metabolism. Annu. Rev. Nutr. 31, 65–87. 10.1146/annurev-nutr-072610-145127 21529158 PMC3789243

[B12] Chassaing N. Golzio C. Odent S. Lequeux L. Vigouroux A. Martinovic-Bouriel J. (2009). Phenotypic Spectrum of STRA6 Mutations: from Matthew-Wood Syndrome to Non-lethal Anophthalmia. Hum. Mutat. 30, E673–E681. 10.1002/humu.21023 19309693

[B13] Chen Y. Huang L. Russo A. F. Solursh M. (1992). Retinoic Acid is Enriched in Hensen's Node and is Developmentally Regulated in the Early Chicken Embryo. Proc. Natl. Acad. Sci. 89, 10056–10059. 10.1073/pnas.89.21.10056 1438194 PMC50276

[B14] Chen Y. Huang L. Solursh M. (1994). A Concentration Gradient of Retinoids in the Early *Xenopus laevis* Embryo. Develop. Biol. 161, 70–76. 10.1006/dbio.1994.1008 7904969

[B15] Chen Y. Pollet N. Niehrs C. Pieler T. (2001). Increased XRALDH2 Activity has a Posteriorizing Effect on the central Nervous System of Xenopus Embryos. Mech. Develop. 101, 91–103. 10.1016/S0925-4773(00)00558-X 11231062

[B16] Cho K. W. Y. Blumberg B. Steinbeisser H. De Robertis E. M. (1991). Molecular Nature of Spemann's Organizer: the Role of the Xenopus Homeobox Gene Goosecoid. Cell 67, 1111–1120. 10.1016/0092-8674(91)90288-a 1684739 PMC3102583

[B17] Christian J. L. Moon R. T. (1993). Interactions between Xwnt-8 and Spemann Organizer Signaling Pathways Generate Dorsoventral Pattern in the Embryonic Mesoderm of Xenopus. Genes Dev. 7, 13–28. 10.1101/gad.7.1.13 8422982

[B18] Clagett-Dame M. DeLuca H. F. (2002). The Role of Vitamin A in Mammalian Reproduction and Embryonic Development. Annu. Rev. Nutr. 22, 347–381. 10.1146/annurev.nutr.22.010402.102745E 12055350

[B19] Clagett-Dame M. Knutson D. (2011). Vitamin A in Reproduction and Development. Nutrients 3, 385–428. 10.3390/nu3040385 22254103 PMC3257687

[B20] Collins M. D. Mao G. E. (1999). Teratology of Retinoids. Annu. Rev. Pharmacol. Toxicol. 39, 399–430. 10.1146/annurev.pharmtox.39.1.399 10331090

[B21] Conlon R. A. Rossant J. (1992). Exogenous Retinoic Acid Rapidly Induces Anterior Ectopic Expression of Murine Hox-2 Genes *In Vivo* . Development 116, 357–368. 10.1242/dev.116.2.357 1363087

[B22] Crabb D. W. Matsumoto M. Chang D. You M. (2004). Overview of the Role of Alcohol Dehydrogenase and Aldehyde Dehydrogenase and Their Variants in the Genesis of Alcohol-Related Pathology. Proc. Nutr. Soc. 63, 49–63. 10.1079/PNS2003327 15099407

[B23] Crandall J. E. Goodman T. McCarthy D. M. Duester G. Bhide P. G. Dräger U. C. (2011). Retinoic Acid Influences Neuronal Migration from the Ganglionic eminence to the Cerebral Cortex. J. Neurochem. 119, 723–735. 10.1111/j.1471-4159.2011.07471.x 21895658 PMC3732058

[B24] Creech Kraft J. Schuh T. Juchau M. R. Kimelman D. (1994). Temporal Distribution, Localization and Metabolism of All-Trans-Retinol, Didehydroretinol and All-Trans-Retinal during Xenopus Development. Biochem. J. 301 (Pt 1), 111–119. 10.1042/bj3010111 8037657 PMC1137150

[B25] Cunningham T. J. Duester G. (2015). Mechanisms of Retinoic Acid Signalling and its Roles in Organ and Limb Development. Nat. Rev. Mol. Cel Biol. 16, 110–123. 10.1038/nrm3932 PMC4636111 25560970

[B26] de Roos K. Sonneveld E. Compaan B. ten Berge D. Durston A. J. van der Saag P. T. (1999). Expression of Retinoic Acid 4-hydroxylase (CYP26) during Mouse and *Xenopus laevis* Embryogenesis. Mech. Develop. 82, 205–211. 10.1016/s0925-4773(99)00016-7 10354487

[B27] Del Campo M. Jones K. L. (2017). A Review of the Physical Features of the Fetal Alcohol Spectrum Disorders. Eur. J. Med. Genet. 60, 55–64. 10.1016/j.ejmg.2016.10.004 27729236

[B28] Deltour L. Ang H. L. Duester G. (1996). Ethanol Inhibition of Retinoic Acid Synthesis as a Potential Mechanism for Fetal Alcohol Syndrome. FASEB J. 10, 1050–1057. 10.1096/fasebj.10.9.8801166 8801166

[B29] Dosch R. Niehrs C. (2000). Requirement for Anti-dorsalizing Morphogenetic Protein in Organizer Patterning. Mech. Develop. 90, 195–203. 10.1016/s0925-4773(99)00245-2 10640703

[B30] Draut H. Liebenstein T. Begemann G. (2019). New Insights into the Control of Cell Fate Choices and Differentiation by Retinoic Acid in Cranial, Axial and Caudal Structures. Biomolecules 9, 860. 10.3390/biom9120860 PMC6995509 31835881

[B31] Duerinckx S. Abramowicz M. (2018). The Genetics of Congenitally Small Brains. Semin. Cel Develop. Biol. 76, 76–85. 10.1016/j.semcdb.2017.09.015 28912110

[B32] Duester G. (1991). A Hypothetical Mechanism for Fetal Alcohol Syndrome Involving Ethanol Inhibition of Retinoic Acid Synthesis at the Alcohol Dehydrogenase Step. Alcohol. Clin. Exp. Res. 15, 568–572. 10.1111/j.1530-0277.1991.tb00562.x 1877746

[B33] Dupé V. Matt N. Garnier J.-M. Chambon P. Mark M. Ghyselinck N. B. (2003). A Newborn Lethal Defect Due to Inactivation of Retinaldehyde Dehydrogenase Type 3 is Prevented by Maternal Retinoic Acid Treatment. Proc. Natl. Acad. Sci. 100, 14036–14041. 10.1073/pnas.2336223100 14623956 PMC283541

[B34] Durston A. J. Timmermans J. P. M. Hage W. J. Hendriks H. F. J. de Vries N. J. Heideveld M. (1989). Retinoic Acid Causes an Anteroposterior Transformation in the Developing central Nervous System. Nature 340, 140–144. 10.1038/340140a0 2739735

[B35] Dyment D. A. Sawyer S. L. Warman-Chardon J. Boycott K. M. (2013). Recent Advances in the Genetic Etiology of Brain Malformations. Curr. Neurol. Neurosci. Rep. 13, 364. 10.1007/s11910-013-0364-1 23793931

[B36] Elsea S. H. Williams S. R. (2011). Smith-Magenis Syndrome: Haploinsufficiency of RAI1 Results in Altered Gene Regulation in Neurological and Metabolic Pathways. Expert Rev. Mol. Med. 13, e14. 10.1017/S1462399411001827 21545756

[B37] Epstein M. Pillemer G. Yelin R. Yisraeli J. K. Fainsod A. (1997). Patterning of the Embryo along the Anterior-Posterior axis: the Role of the Caudal Genes. Development 124, 3805–3814. 10.1242/dev.124.19.3805 9367436

[B38] Faheem M. Naseer M. I. Rasool M. Chaudhary A. G. Kumosani T. A. Ilyas A. M. (2015). Molecular Genetics of Human Primary Microcephaly: an Overview. BMC Med. Genomics 8 (Suppl. 1), S4. 10.1186/1755-8794-8-S1-S4 PMC4315316 25951892

[B39] Fainsod A. Kot-Leibovich H. (2018). Xenopus Embryos to Study Fetal Alcohol Syndrome, a Model for Environmental Teratogenesis. Biochem. Cel Biol. 96, 77–87. 10.1139/bcb-2017-0219 29069552

[B40] Fainsod A. Steinbeisser H. De Robertis E. M. (1994). On the Function of BMP-4 in Patterning the Marginal Zone of the Xenopus Embryo. EMBO J. 13, 5015–5025. 10.1002/j.1460-2075.1994.tb06830.x 7957067 PMC395447

[B41] Fainsod A. Bendelac-Kapon L. Shabtai Y. (2020). Fetal Alcohol Spectrum Disorder: Embryogenesis under Reduced Retinoic Acid Signaling Conditions. Subcell Biochem. 95, 197–225. 10.1007/978-3-030-42282-0_8 32297301

[B42] Fainsod A. Abbou T. Bendelac-Kapon L. Edri T. Pillemer G. (2022). “Fetal Alcohol Spectrum Disorder as a Retinoic Acid Deficiency Syndrome,” in Fetal Alcohol Spectrum Disorder. Advances in Research and Practice. Editors Chudley A. E. Hicks G. G. (New York, NY: Springer Nature).

[B43] Gautam P. Nuñez S. C. Narr K. L. Kan E. C. Sowell E. R. (2014). Effects of Prenatal Alcohol Exposure on the Development of white Matter Volume and Change in Executive Function. NeuroImage Clin. 5, 19–27. 10.1016/j.nicl.2014.05.010 24918069 PMC4050317

[B44] Gautam P. Lebel C. Narr K. L. Mattson S. N. May P. A. Adnams C. M. (2015). Volume Changes and Brain-Behavior Relationships in white Matter and Subcortical gray Matter in Children with Prenatal Alcohol Exposure. Hum. Brain Mapp. 36, 2318–2329. 10.1002/hbm.22772 25711175 PMC4631525

[B45] Ghyselinck N. B. Duester G. (2019). Retinoic Acid Signaling Pathways. Development 146, dev167502. 10.1242/dev.167502 31273085 PMC6633611

[B46] Glinka A. Wu W. Delius H. Monaghan A. P. Blumenstock C. Niehrs C. (1998). Dickkopf-1 is a Member of a New Family of Secreted Proteins and Functions in Head Induction. Nature 391, 357–362. 10.1038/34848 9450748

[B47] Graff J. M. Thies R. S. Song J. J. Celeste A. J. Melton D. A. (1994). Studies with a Xenopus BMP Receptor Suggest that Ventral Mesoderm-Inducing Signals Override Dorsal Signals *In Vivo* . Cell 79, 169–179. 10.1016/0092-8674(94)90409-x 7522972

[B48] Grandel H. Lun K. Rauch G.-J. Rhinn M. Piotrowski T. Houart C. (2002). Retinoic Acid Signalling in the Zebrafish Embryo is Necessary during Pre-segmentation Stages to Pattern the Anterior-Posterior axis of the CNS and to Induce a Pectoral Fin Bud. Development 129, 2851–2865. 10.1242/dev.129.12.2851 12050134

[B49] Guerri C. Bazinet A. Riley E. P. (2009). Foetal Alcohol Spectrum Disorders and Alterations in Brain and Behaviour. Alcohol Alcohol. 44, 108–114. 10.1093/alcalc/agn105 19147799 PMC2724862

[B50] Halilagic A. Zile M. H. Studer M. (2003). A Novel Role for Retinoids in Patterning the Avian Forebrain during Presomite Stages. Development 130, 2039–2050. 10.1242/dev.00423 12668619

[B51] Halilagic A. Ribes V. Ghyselinck N. B. Zile M. H. Dollé P. Studer M. (2007). Retinoids Control Anterior and Dorsal Properties in the Developing Forebrain. Develop. Biol. 303, 362–375. 10.1016/j.ydbio.2006.11.021 17184764

[B52] Hogan B. L. M. Thaller C. Eichele G. (1992). Evidence that Hensen's Node is a Site of Retinoic Acid Synthesis. Nature 359, 237–241. 10.1038/359237a0 1528265

[B53] Hollemann T. Chen Y. Grunz H. Pieler T. (1998). Regionalized Metabolic Activity Establishes Boundaries of Retinoic Acid Signalling. EMBO J. 17, 7361–7372. 10.1093/emboj/17.24.7361 9857192 PMC1171081

[B54] Hoshijima K. Jurynec M. J. Klatt Shaw D. Jacobi A. M. Behlke M. A. Grunwald D. J. (2019). Highly Efficient CRISPR-Cas9-Based Methods for Generating Deletion Mutations and F0 Embryos that Lack Gene Function in Zebrafish. Develop. Cel 51, 645–657. 10.1016/j.devcel.2019.10.004 PMC6891219 31708433

[B55] Hsiau T. Conant D. Rossi N. Maures T. Waite K. Yang J. (2018). Inference of CRISPR Edits from Sanger Trace Data. BioRxiv. 10.1101/251082 35119294

[B56] Huang Y. Winklbauer R. (2018). Cell Migration in the Xenopus Gastrula. WIREs Dev. Biol. 7, e325. 10.1002/wdev.325 29944210

[B57] Inui M. Montagner M. Ben-Zvi D. Martello G. Soligo S. Manfrin A. (2012). Self-regulation of the Head-Inducing Properties of the Spemann Organizer. Proc. Natl. Acad. Sci. USA 109, 15354–15359. 10.1073/pnas.1203000109 22949641 PMC3458350

[B58] Ishibashi H. Matsumura N. Hanafusa H. Matsumoto K. Robertis E. M. D. Kuroda H. (2008). Expression of Siamois and Twin in the Blastula Chordin/Noggin Signaling center is Required for Brain Formation in *Xenopus laevis* Embryos. Mech. Develop. 125, 58–66. 10.1016/j.mod.2007.10.005 PMC2292103 18036787

[B59] Jarmasz J. S. Basalah D. A. Chudley A. E. Del Bigio M. R. (2017). Human Brain Abnormalities Associated with Prenatal Alcohol Exposure and Fetal Alcohol Spectrum Disorder. J. Neuropathol. Exp. Neurol. 76, 813–833. 10.1093/jnen/nlx064 28859338 PMC5901082

[B60] Kaneda T. Motoki J.-y. D. (2012). Gastrulation and Pre-gastrulation Morphogenesis, Inductions, and Gene Expression: Similarities and Dissimilarities between Urodelean and Anuran Embryos. Develop. Biol. 369, 1–18. 10.1016/j.ydbio.2012.05.019 22634398

[B61] Kao K. R. Elinson R. P. (1988). The Entire Mesodermal Mantle Behaves as Spemann's Organizer in Dorsoanterior Enhanced *Xenopus laevis* Embryos. Develop. Biol. 127, 64–77. 10.1016/0012-1606(88)90189-3 3282938

[B62] Kedishvili N. Y. (2013). Enzymology of Retinoic Acid Biosynthesis and Degradation. J. Lipid Res. 54, 1744–1760. 10.1194/jlr.R037028 23630397 PMC3679379

[B63] Kiecker C. Lumsden A. (2012). The Role of Organizers in Patterning the Nervous System. Annu. Rev. Neurosci. 35, 347–367. 10.1146/annurev-neuro-062111-150543 22462542

[B64] Kin Ting Kam R. Deng Y. Chen Y. Zhao H. (2012). Retinoic Acid Synthesis and Functions in Early Embryonic Development. Cell Biosci. 2, 11. 10.1186/2045-3701-2-11 22439772 PMC3325842

[B65] Koide T. Downes M. Chandraratna R. A. S. Blumberg B. Umesono K. (2001). Active Repression of RAR Signaling is Required for Head Formation. Genes Dev. 15, 2111–2121. 10.1101/gad.908801 11511542 PMC312762

[B66] Koide T. Umesono K. Hashimoto C. (2002). When Does the Anterior Endomesderm Meet the Anterior-Most Neuroectoderm during Xenopus Gastrulation? Int. J. Dev. Biol. 46, 777–783. 12382943

[B67] Kot-Leibovich H. Fainsod A. (2009). Ethanol Induces Embryonic Malformations by Competing for Retinaldehyde Dehydrogenase Activity during Vertebrate Gastrulation. Dis. Model. Mech. 2, 295–305. 10.1242/dmm.001420 19380308 PMC2675815

[B68] Koyabu D. Werneburg I. Morimoto N. Zollikofer C. P. E. Forasiepi A. M. Endo H. (2014). Mammalian Skull Heterochrony Reveals Modular Evolution and a Link between Cranial Development and Brain Size. Nat. Commun. 5, 3625. 10.1038/ncomms4625 24704703 PMC3988809

[B69] Kraft J. C. Schuh T. Juchau M. Kimelman D. (1994). The Retinoid X Receptor Ligand, 9-Cis-Retinoic Acid, is a Potential Regulator of Early Xenopus Development. Proc. Natl. Acad. Sci. 91, 3067–3071. 10.1073/pnas.91.8.3067 8159708 PMC43516

[B70] Krieg P. A. Sakaguchi D. S. Kintner C. R. (1989). Primary Structure and Developmental Expression of a Large Cytoptasmic Domain Form of Xenopus Laevisneural Cell Adhesion Molecule (NCAM). Nucl. Acids Res. 17, 10321–10335. 10.1093/nar/17.24.10321 2481269 PMC335303

[B71] Kuroda H. Wessely O. Robertis E. M. D. (2004). Neural Induction in Xenopus: Requirement for Ectodermal and Endomesodermal Signals *via* Chordin, Noggin, *β*-Catenin, and Cerberus. Plos Biol. 2, E92. 10.1371/journal.pbio.0020092 15138495 PMC406387

[B72] Li H. Tierney C. Wen L. Wu J. Y. Rao Y. (1997). A Single Morphogenetic Field Gives Rise to Two Retina Primordia under the Influence of the Prechordal Plate. Development 124, 603–615. 10.1242/dev.124.3.603 9043075 PMC2041934

[B73] Liang D. Zhang M. Bao J. Zhang L. Xu X. Gao X. (2008). Expressions of Raldh3 and Raldh4 during Zebrafish Early Development. Gene Expr. Patterns 8, 248–253. 10.1016/j.gep.2007.12.007 18262854

[B74] Lloret-Vilaspasa F. Jansen H. J. Deroos K. Chandraratna R. A. S. Zile M. H. Stern C. D. (2010). Retinoid Signalling is Required for Information Transfer from Mesoderm to Neuroectoderm during Gastrulation. Int. J. Dev. Biol. 54, 599–608. 10.1387/ijdb.082705fl 20209433

[B75] Lohnes D. Mark M. Mendelsohn C. Dollé P. Dierich A. Gorry P. (1994). Function of the Retinoic Acid Receptors (RARs) during Development (I). Craniofacial and Skeletal Abnormalities in RAR Double Mutants. Development 120, 2723–2748. 10.1242/dev.120.10.2723 7607067

[B76] Lupo G. Liu Y. Qiu R. Chandraratna R. A. S. Barsacchi G. He R.-Q. (2005). Dorsoventral Patterning of the Xenopus Eye: a Collaboration of Retinoid, Hedgehog and FGF Receptor Signaling. Development 132, 1737–1748. 10.1242/dev.01726 15753216

[B77] Maden M. (2000). The Role of Retinoic Acid in Embryonic and post-embryonic Development. Proc. Nutr. Soc. 59, 65–73. 10.1017/s0029665100000082 10828175

[B78] Mark M. Ghyselinck N. B. Chambon P. (2006). Function of Retinoid Nuclear Receptors: Lessons from Genetic and Pharmacological Dissections of the Retinoic Acid Signaling Pathway during Mouse Embryogenesis. Annu. Rev. Pharmacol. Toxicol. 46, 451–480. 10.1146/annurev.pharmtox.46.120604.141156 16402912

[B79] Mark M. Ghyselinck N. B. Chambon P. (2009). Function of Retinoic Acid Receptors during Embryonic Development. Nucl. Recept. Signal. 7, nrs.07002. 10.1621/nrs.07002 PMC2670431 19381305

[B80] Marom K. Levy V. Pillemer G. Fainsod A. (2005). Temporal Analysis of the Early BMP Functions Identifies Distinct Anti-organizer and Mesoderm Patterning Phases. Develop. Biol. 282, 442–454. 10.1016/j.ydbio.2005.03.024 15950609

[B81] Martini M. Klausing A. Lüchters G. Heim N. Messing-Jünger M. (2018). Head Circumference - a Useful Single Parameter for Skull Volume Development in Cranial Growth Analysis? Head Face Med. 14, 3. 10.1186/s13005-017-0159-8 29321071 PMC5764008

[B82] Matsuo I. Kuratani S. Kimura C. Takeda N. Aizawa S. (1995). Mouse Otx2 Functions in the Formation and Patterning of Rostral Head. Genes Dev. 9, 2646–2658. 10.1101/gad.9.21.2646 7590242

[B83] Mendelsohn C. Lohnes D. Décimo D. Lufkin T. LeMeur M. Chambon P. (1994). Function of the Retinoic Acid Receptors (RARs) during Development (II). Multiple Abnormalities at Various Stages of Organogenesis in RAR Double Mutants. Development 120, 2749–2771. 10.1242/dev.120.10.2749 7607068

[B84] Mic F. A. Molotkov A. Molotkova N. Duester G. (2004). Raldh2 Expression in Optic Vesicle Generates a Retinoic Acid Signal Needed for Invagination of Retina during Optic Cup Formation. Dev. Dyn. 231, 270–277. 10.1002/dvdy.20128 15366004

[B85] Mochida G. H. (2009). Genetics and Biology of Microcephaly and Lissencephaly. Semin. Pediatr. Neurol. 16, 120–126. 10.1016/j.spen.2009.07.001 19778709 PMC3565221

[B86] Molotkova N. Molotkov A. Duester G. (2007). Role of Retinoic Acid during Forebrain Development Begins Late when Raldh3 Generates Retinoic Acid in the Ventral Subventricular Zone. Develop. Biol. 303, 601–610. 10.1016/j.ydbio.2006.11.035 17207476 PMC1994967

[B87] Moreno-Mateos M. A. Vejnar C. E. Beaudoin J.-D. Fernandez J. P. Mis E. K. Khokha M. K. (2015). CRISPRscan: Designing Highly Efficient sgRNAs for CRISPR-Cas9 Targeting *In Vivo* . Nat. Methods 12, 982–988. 10.1038/nmeth.3543 26322839 PMC4589495

[B88] Morgan C. A. Parajuli B. Buchman C. D. Dria K. Hurley T. D. (2015). N,N-diethylaminobenzaldehyde (DEAB) as a Substrate and Mechanism-Based Inhibitor for Human ALDH Isoenzymes. Chem. Biol. Interact. 234, 18–28. 10.1016/j.cbi.2014.12.008 25512087 PMC4414715

[B89] Muralidharan P. Sarmah S. Marrs J. A. (2015). Zebrafish Retinal Defects Induced by Ethanol Exposure are Rescued by Retinoic Acid and Folic Acid Supplement. Alcohol 49, 149–163. 10.1016/j.alcohol.2014.11.001 25541501 PMC4339401

[B90] Naert T. Tulkens D. Edwards N. A. Carron M. Shaidani N.-I. Wlizla M. (2020). Maximizing CRISPR/Cas9 Phenotype Penetrance Applying Predictive Modeling of Editing Outcomes in Xenopus and Zebrafish Embryos. Sci. Rep. 10, 14662. 10.1038/s41598-020-71412-0 32887910 PMC7473854

[B91] Naito Y. Hino K. Bono H. Ui-Tei K. (2015). CRISPRdirect: Software for Designing CRISPR/Cas Guide RNA with Reduced Off-Target Sites. Bioinformatics 31, 1120–1123. 10.1093/bioinformatics/btu743 25414360 PMC4382898

[B92] Nakatsuji N. (1983). Craniofacial Malformation inXenopus Laevis Tadpoles Caused by the Exposure of Early Embryos to Ethanol. Teratology 28, 299–305. 10.1002/tera.1420280220 6648833

[B93] Natale V. Rajagopalan A. (2014). Worldwide Variation in Human Growth and the World Health Organization Growth Standards: a Systematic Review. BMJ Open 4, e003735. 10.1136/bmjopen-2013-003735 PMC3902406 24401723

[B94] Nenni M. J. Fisher M. E. James-Zorn C. Pells T. J. Ponferrada V. Chu S. (2019). Xenbase: Facilitating the Use of Xenopus to Model Human Disease. Front. Physiol. 10, 154. 10.3389/fphys.2019.00154 30863320 PMC6399412

[B95] Niccols A. (2007). Fetal Alcohol Syndrome and the Developing Socio-Emotional Brain. Brain Cogn. 65, 135–142. 10.1016/j.bandc.2007.02.009 17669569

[B96] Niederreither K. McCaffery P. Dräger U. C. Chambon P. Dollé P. (1997). Restricted Expression and Retinoic Acid-Induced Downregulation of the Retinaldehyde Dehydrogenase Type 2 (RALDH-2) Gene during Mouse Development. Mech. Develop. 62, 67–78. 10.1016/S0925-4773(96)00653-3 9106168

[B97] Niederreither K. Subbarayan V. Dollé P. Chambon P. (1999). Embryonic Retinoic Acid Synthesis is Essential for Early Mouse post-implantation Development. Nat. Genet. 21, 444–448. 10.1038/7788 10192400

[B98] Niehrs C. Kazanskaya O. Wu W. Glinka A. (2001). Dickkopf1 and the Spemann-Mangold Head Organizer. Int. J. Dev. Biol. 45, 237–240. 11291852

[B99] Niehrs C. (2004). Regionally Specific Induction by the Spemann-Mangold Organizer. Nat. Rev. Genet. 5, 425–434. 10.1038/nrg1347 15153995

[B100] Nieuwkoop P. D. Faber J. (1967). Normal Table of *Xenopus laevis* (Daudin): A Systematical and Chronological Survey of the Development from the Fertilized Egg till the End of Metamorphosis. Amsterdam: North-Holland Publishing Company.

[B101] Nolte C. De Kumar B. Krumlauf R. (2019). Hox Genes: Downstream “Effectors” of Retinoic Acid Signaling in Vertebrate Embryogenesis. Genesis 57, e23306. 10.1002/dvg.23306 31111645

[B150] Pannese M. Polo C. Andreazzoli M. Vignali R. Kablar B. Barsacchi G. (1995). The Xenopus homologue of Otx2 is a maternal homeobox gene that demarcates and specifies anterior body regions. Development 121, 707–720. 10.1242/dev.121.3.707 7720578

[B102] Parihar M. Bendelac-Kapon L. Gur M. Abbou T. Belorkar A. Achanta S. (2021). Retinoic Acid Fluctuation Activates an Uneven, Direction-dependent Network-wide Robustness Response in Early Embryogenesis. Front. Cel Dev. Biol. 9, 747969. 10.3389/fcell.2021.747969 PMC8564372 34746144

[B103] Perz-Edwards A. Hardison N. L. Linney E. (2001). Retinoic Acid-Mediated Gene Expression in Transgenic Reporter Zebrafish. Develop. Biol. 229, 89–101. 10.1006/dbio.2000.9979 11133156

[B104] Petrelli B. Bendelac L. Hicks G. G. Fainsod A. (2019). Insights into Retinoic Acid Deficiency and the Induction of Craniofacial Malformations and Microcephaly in Fetal Alcohol Spectrum Disorder. Genesis 57, e23278. 10.1002/dvg.23278 30614633

[B105] Popova S. Lange S. Shield K. Mihic A. Chudley A. E. Mukherjee R. A. S. (2016). Comorbidity of Fetal Alcohol Spectrum Disorder: a Systematic Review and Meta-Analysis. The Lancet 387, 978–987. 10.1016/S0140-6736(15)01345-8 26777270

[B106] Pullarkat R. K. (1991). Hypothesis: Prenatal Ethanol-Induced Birth Defects and Retinoic Acid. Alcohol. Clin. Exp. Res. 15, 565–567. 10.1111/j.1530-0277.1991.tb00561.x 1877745

[B107] Ranke M. B. Krägeloh-Mann I. Vollmer B. (2015). Growth, Head Growth, and Neurocognitive Outcome in Children Born Very Preterm: Methodological Aspects and Selected Results. Dev. Med. Child. Neurol. 57, 23–28. 10.1111/dmcn.12582 25251724

[B108] Rhinn M. Dollé P. (2012). Retinoic Acid Signalling during Development. Development 139, 843–858. 10.1242/dev.065938 22318625

[B109] Rhinn M. Schuhbaur B. Niederreither K. Dollé P. (2011). Involvement of Retinol Dehydrogenase 10 in Embryonic Patterning and rescue of its Loss of Function by Maternal Retinaldehyde Treatment. Proc. Natl. Acad. Sci. 108, 16687–16692. 10.1073/pnas.1103877108 21930923 PMC3189044

[B110] Ribes V. Wang Z. Dollé P. Niederreither K. (2006). Retinaldehyde Dehydrogenase 2 (RALDH2)-Mediated Retinoic Acid Synthesis Regulates Early Mouse Embryonic Forebrain Development by Controlling FGF and Sonic Hedgehog Signaling. Development 133, 351–361. 10.1242/dev.02204 16368932

[B111] Ribes V. Fraulob V. Petkovich M. Dollé P. (2007). The Oxidizing Enzyme CYP26a1 Tightly Regulates the Availability of Retinoic Acid in the Gastrulating Mouse Embryo to Ensure Proper Head Development and Vasculogenesis. Dev. Dyn. 236, 644–653. 10.1002/dvdy.21057 17211890

[B112] Ross S. A. McCaffery P. J. Drager U. C. De Luca L. M. (2000). Retinoids in Embryonal Development. Physiol. Rev. 80, 1021–1054. 10.1152/physrev.2000.80.3.1021 10893430

[B113] Rossant J. Zirngibl R. Cado D. Shago M. Giguère V. (1991). Expression of a Retinoic Acid Response Element-hsplacZ Transgene Defines Specific Domains of Transcriptional Activity during Mouse Embryogenesis. Genes Dev. 5, 1333–1344. 10.1101/gad.5.8.1333 1907940

[B114] Roussotte F. F. Sulik K. K. Mattson S. N. Riley E. P. Jones K. L. Adnams C. M. (2012). Regional Brain Volume Reductions Relate to Facial Dysmorphology and Neurocognitive Function in Fetal Alcohol Spectrum Disorders. Hum. Brain Mapp. 33, 920–937. 10.1002/hbm.21260 21416562 PMC3812802

[B115] Russo J. E. Hauquitz D. Hilton J. (1988). Inhibition of Mouse Cytosolic Aldehyde Dehydrogenase by 4-(diethylamino) benzaldehyde. Biochem. Pharmacol. 37, 1639–1642. 10.1016/0006-2952(88)90030-5 3358794

[B116] Samarut E. Fraher D. Laudet V. Gibert Y. (2015). ZebRA: An Overview of Retinoic Acid Signaling during Zebrafish Development. Biochim. Biophys. Acta Gene Regul. Mech. 1849, 73–83. 10.1016/j.bbagrm.2014.05.030 24928143

[B117] Sandell L. L. Sanderson B. W. Moiseyev G. Johnson T. Mushegian A. Young K. (2007). RDH10 is Essential for Synthesis of Embryonic Retinoic Acid and is Required for Limb, Craniofacial, and Organ Development. Genes Dev. 21, 1113–1124. 10.1101/gad.1533407 17473173 PMC1855236

[B118] Sasai Y. Lu B. Steinbeisser H. Geissert D. Gont L. K. De Robertis E. M. (1994). Xenopus Chordin: a Novel Dorsalizing Factor Activated by Organizer-specific Homeobox Genes. Cell 79, 779–790. 10.1016/0092-8674(94)90068-x 8001117 PMC3082463

[B119] See A. W.-M. Kaiser M. E. White J. C. Clagett-Dame M. (2008). A Nutritional Model of Late Embryonic Vitamin A Deficiency Produces Defects in Organogenesis at a High Penetrance and Reveals New Roles for the Vitamin in Skeletal Development. Develop. Biol. 316, 171–190. 10.1016/j.ydbio.2007.10.018 18321479

[B120] Shabtai Y. Fainsod A. (2018). Competition between Ethanol Clearance and Retinoic Acid Biosynthesis in the Induction of Fetal Alcohol Syndrome. Biochem. Cel Biol. 96, 148–160. 10.1139/bcb-2017-0132 28982012

[B121] Shabtai Y. Jubran H. Nassar T. Hirschberg J. Fainsod A. (2016). Kinetic Characterization and Regulation of the Human Retinaldehyde Dehydrogenase 2 Enzyme during Production of Retinoic Acid. Biochem. J. 473, 1423–1431. 10.1042/BCJ20160101 27001866

[B122] Shabtai Y. Bendelac L. Jubran H. Hirschberg J. Fainsod A. (2018). Acetaldehyde Inhibits Retinoic Acid Biosynthesis to Mediate Alcohol Teratogenicity. Sci. Rep. 8, 347. 10.1038/s41598-017-18719-7 29321611 PMC5762763

[B123] Shen M. W. Arbab M. Hsu J. Y. Worstell D. Culbertson S. J. Krabbe O. (2018). Predictable and Precise Template-free CRISPR Editing of Pathogenic Variants. Nature 563, 646–651. 10.1038/s41586-018-0686-x 30405244 PMC6517069

[B124] Shiotsugu J. Katsuyama Y. Arima K. Baxter A. Koide T. Song J. (2004). Multiple Points of Interaction between Retinoic Acid and FGF Signaling during Embryonic Axis Formation. Development 131, 2653–2667. 10.1242/dev.01129 15128657

[B125] Shukrun N. Shabtai Y. Pillemer G. Fainsod A. (2019). Retinoic Acid Signaling Reduction Recapitulates the Effects of Alcohol on Embryo Size. Genesis 57, e23284. 10.1002/dvg.23284 30672660

[B126] Sive H. L. Hattori K. Weintraub H. (1989). Progressive Determination during Formation of the Anteroposterior axis in *Xenopus laevis* . Cell 58, 171–180. 10.1016/0092-8674(89)90413-3 2752418

[B127] Sive H. L. Draper B. W. Harland R. M. Weintraub H. (1990). Identification of a Retinoic Acid-Sensitive Period during Primary axis Formation in *Xenopus laevis* . Genes Dev. 4, 932–942. 10.1101/gad.4.6.932 2384214

[B128] Smith W. C. Knecht A. K. Wu M. Harland R. M. (1993). Secreted Noggin Protein Mimics the Spemann Organizer in Dorsalizing Xenopus Mesoderm. Nature 361, 547–549. 10.1038/361547a0 8429909

[B129] Sokol S. Christian J. L. Moon R. T. Melton D. A. (1991). Injected Wnt RNA Induces a Complete Body axis in Xenopus Embryos. Cell 67, 741–752. 10.1016/0092-8674(91)90069-b 1834344

[B130] Spemann H. Mangold H. (1924). über Induktion von Embryonalanlagen durch Implantation artfremder Organisatoren. Archiv F Mikr Anat. U Entwicklungsmechanik 100, 599–638. 10.1007/bf02108133

[B131] Spohr H.-L. Steinhausen H.-C. (2008). Fetal Alcohol Spectrum Disorders and their Persisting Sequelae in Adult Life. Dtsch Arztebl Int. 105, 693–698. 10.3238/arztebl.2008.0693 19623288 PMC2696967

[B132] Strate I. Min T. H. Iliev D. Pera E. M. (2009). Retinol Dehydrogenase 10 is a Feedback Regulator of Retinoic Acid Signalling during axis Formation and Patterning of the central Nervous System. Development 136, 461–472. 10.1242/dev.024901 19141675

[B133] Tanaka S. Hosokawa H. Weinberg E. S. Maegawa S. (2017). Chordin and Dickkopf-1b are Essential for the Formation of Head Structures through Activation of the FGF Signaling Pathway in Zebrafish. Develop. Biol. 424, 189–197. 10.1016/j.ydbio.2017.02.018 28259755

[B134] Tandon P. Conlon F. Furlow J. D. Horb M. E. (2017). Expanding the Genetic Toolkit in Xenopus : Approaches and Opportunities for Human Disease Modeling. Develop. Biol. 426, 325–335. 10.1016/j.ydbio.2016.04.009 27109192 PMC5074924

[B135] Tanibe M. Michiue T. Yukita A. Danno H. Ikuzawa M. Ishiura S. (2008). Retinoic Acid Metabolizing Factor xCyp26c is Specifically Expressed in Neuroectoderm and Regulates Anterior Neural Patterning in *Xenopus laevis* . Int. J. Dev. Biol. 52, 893–901. 10.1387/ijdb.082683mt 18956319

[B136] Tanibe M. Ishiura S.-I. Asashima M. Michiue T. (2012). xCOUP-TF-B Regulates xCyp26 Transcription and Modulates Retinoic Acid Signaling for Anterior Neural Patterning in Xenopus. Int. J. Dev. Biol. 56, 239–244. 10.1387/ijdb.113482mt 22562199

[B137] Toi A. Chitayat D. Blaser S. (2009). Abnormalities of the Foetal Cerebral Cortex. Prenat. Diagn. 29, 355–371. 10.1002/pd.2211 19235759

[B138] Treit S. Chen Z. Zhou D. Baugh L. Rasmussen C. Andrew G. (2017). Sexual Dimorphism of Volume Reduction but Not Cognitive Deficit in Fetal Alcohol Spectrum Disorders: A Combined Diffusion Tensor Imaging, Cortical Thickness and Brain Volume Study. NeuroImage Clin. 15, 284–297. 10.1016/j.nicl.2017.05.006 28560153 PMC5440763

[B139] Twal W. Roze L. Zile M. H. (1995). Anti-retinoic Acid Monoclonal Antibody Localizes All-Trans-Retinoic Acid in Target Cells and Blocks normal Development in Early Quail Embryo. Develop. Biol. 168, 225–234. 10.1006/dbio.1995.1075 7729565

[B140] Ulven S. M. Gundersen T. E. Weedon M. S. Landaas V. Ø. Sakhi A. K. Fromm S. H. (2000). Identification of Endogenous Retinoids, Enzymes, Binding Proteins, and Receptors during Early Postimplantation Development in Mouse: Important Role of Retinal Dehydrogenase Type 2 in Synthesis of All-Trans-Retinoic Acid. Develop. Biol. 220, 379–391. 10.1006/dbio.2000.9634 10753524

[B141] Vermot J. Niederreither K. Garnier J.-M. Chambon P. Dollé P. (2003). Decreased Embryonic Retinoic Acid Synthesis Results in a DiGeorge Syndrome Phenotype in Newborn Mice. Proc. Natl. Acad. Sci. 100, 1763–1768. 10.1073/pnas.0437920100 12563036 PMC149907

[B142] Weston A. D. Blumberg B. Underhill T. M. (2003). Active Repression by Unliganded Retinoid Receptors in Development. J. Cel Biol. 161, 223–228. 10.1083/jcb.200211117 PMC2172895 12719467

[B143] Whiting J. (1997). Craniofacial Abnormalities Induced by the Ectopic Expression of Homeobox Genes. Mutat. Res. Fund. Mol. Mech. Mutagen. 396, 97–112. 10.1016/s0027-5107(97)00177-2 9434862

[B144] Yanagi T. Ito K. Nishihara A. Minamino R. Mori S. Sumida M. (2015). The S Pemann Organizer Meets the Anterior‐most Neuroectoderm at the Equator of Early Gastrulae in Amphibian Species. Develop. Growth Differ. 57, 218–231. 10.1111/dgd.12200 PMC4402005 25754292

[B145] Yelin R. Ben-Haroush Schyr R. Kot H. Zins S. Frumkin A. Pillemer G. (2005). Ethanol Exposure Affects Gene Expression in the Embryonic Organizer and Reduces Retinoic Acid Levels. Develop. Biol. 279, 193–204. 10.1016/j.ydbio.2004.12.014 15708568

[B146] Yelin R. Kot H. Yelin D. Fainsod A. (2007). Early Molecular Effects of Ethanol during Vertebrate Embryogenesis. Differentiation 75, 393–403. 10.1111/j.1432-0436.2006.00147.x 17286601

[B147] Zaffran S. Odelin G. Stefanovic S. Lescroart F. Etchevers H. C. (2018). Ectopic Expression of Hoxb1 Induces Cardiac and Craniofacial Malformations. Genesis 56, e23221. 10.1002/dvg.23221 30134070

[B148] Zhong G. Hogarth C. Snyder J. M. Palau L. Topping T. Huang W. (2019). The Retinoic Acid Hydroxylase Cyp26a1 Has Minor Effects on Postnatal Vitamin A Homeostasis, but is Required for Exogenous atRA Clearance. J. Biol. Chem. 294, 11166–11179. 10.1074/jbc.RA119.009023 31167781 PMC6643038

