# Supplementary material for: Reduced Retinoic Acid Signaling During Gastrulation Induces Developmental Microcephaly
Source: Front Cell Dev Biol. 2022 Mar 14;10:844619. doi: 10.3389/fcell.2022.844619 (PMC8967241; doi:10.3389/fcell.2022.844619)
Supplement: Supplementary file 1 [file DataSheet1.PDF]

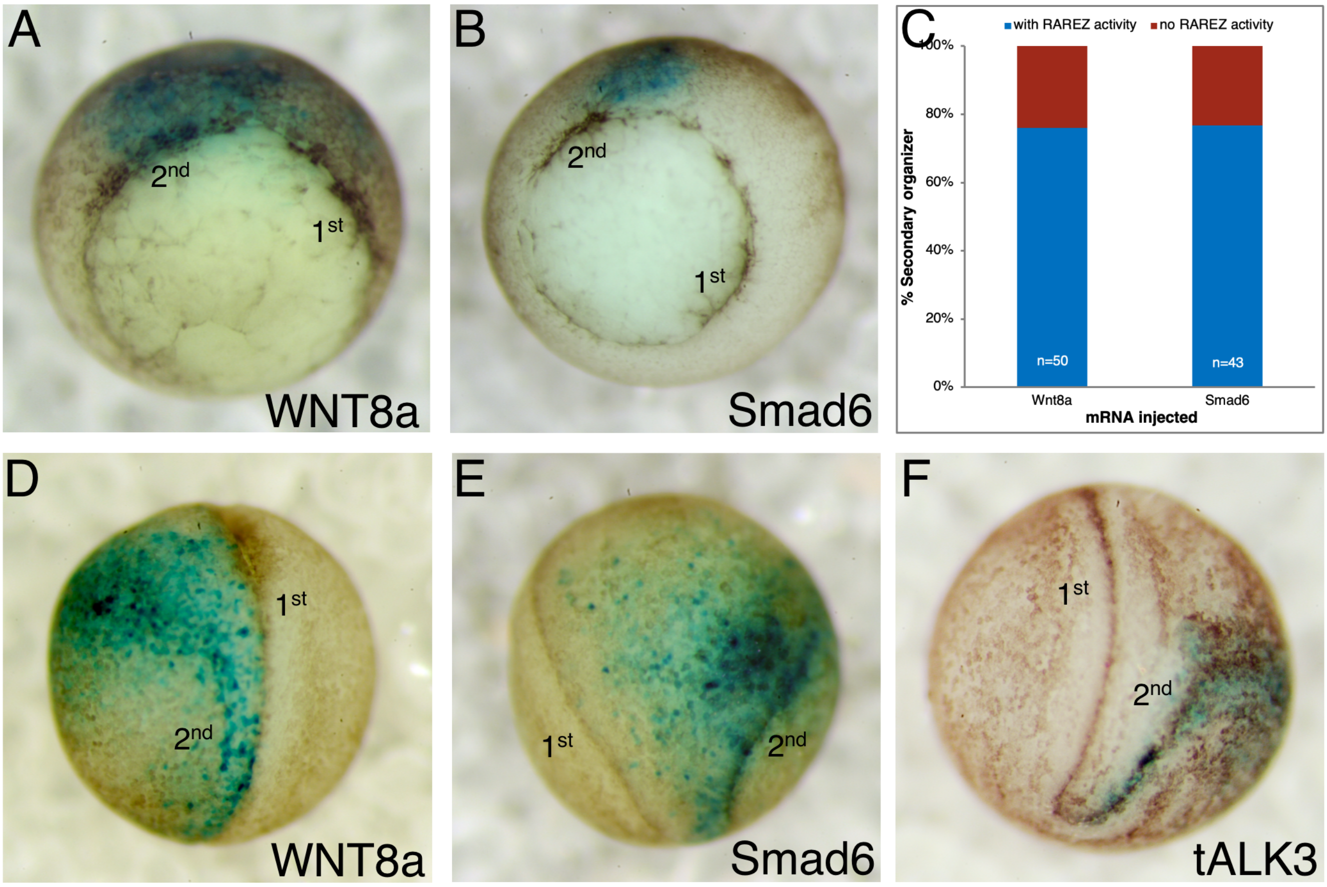

Supplemental Figure S1. RA signaling is active in induced secondary axes. Embryos were injected ventrally with *wnt8a* (A,D), *smad6* (B,E), or dominant negative BMP receptor (tALK3)(F). RNA to activate Wnt/ß-catenin or inhibit BMP signaling and induce secondary axes. The RA reporter plasmid, RAREZ, was co-injected with the RNA to detect active RA signaling in the induced secondary axis. Injected embryos were stained for ß-galactosidase activity during early gastrula (st. 10.25) (A,B) or early neurula (st. 14/15)(D-F). The primary and secondary axes are labeled (1st, 2nd). (C) The percentage of embryos with two dorsal blastopore lips exhibiting or lacking ß-galactosidase staining from the RAREZ reporter plasmid was determined.

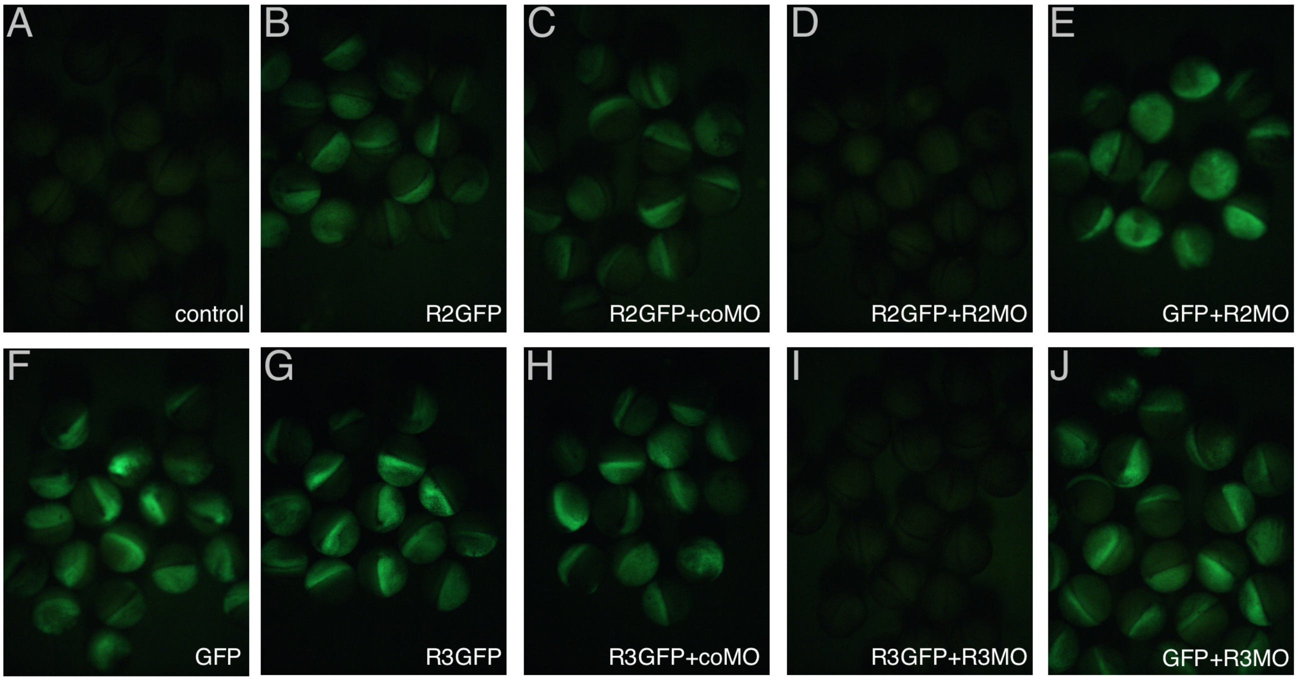

Supplemental Figure S2. Antisense morpholino oligonucleotides against *aldh1a3* and *aldh1a2*. Embryos at the 2-cell stage were injected in one of the cells with RNA encoding either GFP, GFP preceded by the *aldh1a2* morpholino target site (R2GFP), or GFP preceded by the *aldh1a3* morpholino target site (R3GFP). These RNAs were co-injected with either control morpholino oligonucleotide (coMO), or morpholinos targeting the *aldh1a2* (R2MO) or *aldh1a3* (R3MO) transcripts. (A) Control uninjected embryos. (B) R2GFP, (C) R2GFP+coMO, (D) R2GFP+R2MO, (E) GFP+R2MO, (F) GFP, (G) R3GFP, (H) R3GFP+coMO, (I) R3GFP+R3MO, (J) GFP+R3MO.

A

aldh1a2.L	TCTGTGTGGAGGAGAATGGATGCCTCAGAAAGGGGGCGGCTGCTGGATAAATTGGCTGAT
aldh1a2.S	TCTGTGTGGAGGAGAATGGATGCCTCAGAAAGGGGGCGACTGCTGGATAAATTAGCCGAT
aldh1a3.L	TCTGCGTGGAGACGGTTGGATGCCAATGGCCGAGGGAGACTGCTGCACAAACTGGGAGAC
aldh1a3.S	TCTGCGTGGAGAAGGCTGGATGCCAATGGCCGAGGGAGACTGCTACACAAACTTGGAGAC

B

ALDH1A2.L	SVWRRMDASERGRLLDKLAD
ALDH1A2.S	SVWRRMDASERGRLLDKLAD
ALDH1A3.L	SAWRRLDANGRGRLLHKLGD
ALDH1A3.S	SAWRRLDANGRGRLLHKLGD

C

Frameshift efficiency:

Gene	PAM	INDEL [%]	R^2	Knockout score
aldh1a2.L	GGG	97	0.97	95
aldh1a2.S	GGG	92	0.94	92
aldh1a3.L	TGG	91	0.97	78
aldh1a3.S	TGG	71	0.96	71

D

E

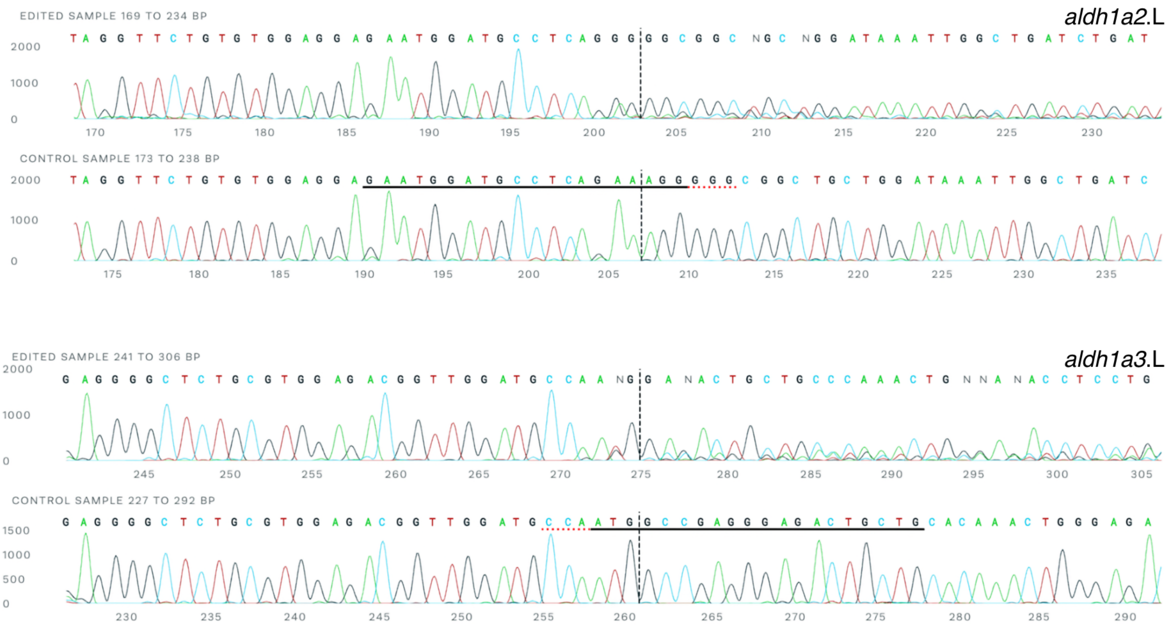

Supplementary Figure S3. Targeting the *aldh1a2* and *aldh1a3* genes with CRISPR/Cas9. To generate *aldh1a2* and *aldh1a3* CRISPant embryos we designed sgRNAs that recognize both homoeologs of each gene in regions with low homology between these two genes. (B) The targeted regions include non-conserved domains between the ALDH1A2 and ALDH1A3 proteins. (C-E) Validation of the indel formation using CRISPR/Cas9 was obtained by nested PCR amplification of genomic DNA from CRISPant and control embryos, followed by sequencing and decomposition analysis. (C) Summary of the indel and frameshift efficiencies from the decomposition analysis of both homoeologs of *aldh1a2* and *aldh1a3*. (D-E) Sample sequencing traces of *aldh1a2*.L and *aldh1a3*.L with the CRISPant DNA above and the control sibling DNA below. The position of the sgRNA (black line) and the PAM sequence (red dotted line) are marked.
